# The Application of NMR Spectroscopy and Chemometrics in Authentication of Spices

**DOI:** 10.3390/molecules26020382

**Published:** 2021-01-13

**Authors:** Barbara Pacholczyk-Sienicka, Grzegorz Ciepielowski, Łukasz Albrecht

**Affiliations:** Institute of Organic Chemistry, Faculty of Chemistry, Lodz University of Technology, Zeromskiego 116, 90-924 Lodz, Poland; grzegorz.ciepielowski@p.lodz.pl (G.C.); lukasz.albrecht@p.lodz.pl (Ł.A.)

**Keywords:** spices adulteration, NMR spectroscopy, chemometrics

## Abstract

Spices and herbs are among the most commonly adulterated food types. This is because spices are widely used to process food. Spices not only enhance the flavor and taste of food, but they are also sources of numerous bioactive compounds that are significantly beneficial for health. The healing effects of spices are connected with their antimicrobial, anti-inflammatory and carminative properties. However, regular consumption of adulterated spices may cause fatal damage to our system because adulterants in most cases are unhealthy. For that reason, the appropriate analytical methods are necessary for quality assurance and to ensure the authenticity of spices. Spectroscopic methods are gaining interest as they are fast, require little or no sample preparation, and provide rich structural information. This review provides an overview of the application of NMR spectroscopy combined with chemometric analysis to determine the quality and adulteration of spices.

## 1. Introduction

In 2019, the global seasoning and spices market was valued at about 13.8 billion U.S. dollars, and is expected to grow annually by 6.3% (Compound Annual Growth Rate, CAGR 2020–2027). It is caused by the change of lifestyles, growing consumer preference for ready-made spices in order to save time and their inclination towards ethnic cuisines [1,2]. The globalization of spices trade and its high demand in cuisines of every country leads to the expansion of condiments counterfeiting. Moreover, spices are particularly susceptible to falsification because they are sold in powder form and have a long and complicated supply chain [3]. According to a Europol report from 2019, about 16,000 tons and 33 million liters of potentially dangerous fake food and drink was seized. Spices constituted about 1136 metric tons. The overall value of counterfeit food and drink was estimated at over 100 million EUR [4]. The illegal market of food counterfeiting is huge and causes a loss of approximately 49 billion USD per year [5].

By definition “food fraud is an intentional substitution, addition, tampering, or misrepresentation of food, food ingredients, or food packaging, or false or misleading statements made about a product for economic gain” [6]. According to the Food Ingredients Expert Committee, all these manipulations are made without the purchaser’s knowledge [7,8,9,10,11]. To describe food fraud, terms such as economic adulteration, food counterfeiting or economically motivated adulteration are used, but food fraud is a broader term than the others. Three main types of food fraud can be distinguished: (1) replacement, (2) addition, and (3) removal. The first type includes complete or partial replacement of a food ingredient or valuable authentic constituent with a less expensive substitute. This kind of adulteration can be achieved by the addition, dilution, or extension of an authentic ingredient with an adulterant or mixture of adulterants, for example addition of melamine to milk or wheat gluten to artificially increase apparent protein contents or over-glazing frozen fish (adds extra water). Moreover, the replacement type includes three subtypes: (1) false declaration of geographical, botanical and varietal origin, for example the replacement of Italian olive oil with Greek olive oil, and more expensive goat or sheep’s milk with cow’s milk, (2) false declaration of the raw material origin or production process, such as substitution of synthetically produced vanillin for botanically derived natural vanillin, and (3) false declaration of origin to evade taxes or tariffs as in the case of Chinese shrimp, which are transshipped from Indonesia to avoid antidumping duties [7,10,12]. The second type refers to addition of small amounts of a non-authentic substance to mask an inferior quality ingredient. Examples include both color and taste enhancement: addition of dyes to make a spice look fresher and sugar to mask the astringent taste of poor-quality pomegranate juice [7,13,14]. The third type of food fraud is based on the removal or intentional omission of an authentic and valuable constituent in a food product or food ingredient. For examples the removal of non-polar constituents from paprika (such as lipids and flavor compounds) to produce paprika-derived flavoring extracts or removal of pollen and other residue from the honey in order to make it difficult to determine the honey’s botanical and geographical origin [7,8].

As a consequence of the mentioned practices, consumers buy products of reduced value that contain substances that do not have their presence legally declared, are not permitted or are present in a form which might mislead the consumer. The problem of food counterfeiting is worldwide and has resulted in widely reported an international food safety incidents [7,9,10,13,15]. For example, in 1994 consumption of paprika powder adulterated with lead oxide caused 60 hospitalizations in Hungary, and nearly 300,000 children became ill and 6 children died in China in 2008 from melamine adulteration of infant formula [10,11]. Since 2003, numerous notifications reported to the EU rapid alert system for food and feed (RASFF) have demonstrated the illegal presence of Sudan and Para red dyes in a range of foodstuffs including: chili and chili products (Sudan I and IV, Para Red), curry and curry products (Sudan I), sumac (Sudan I and IV), curcuma (Sudan I and Sudan IV), saffron (Sudan I and IV) and palm oil (Sudan IV). It is worthy to mention that the International Agency for Research on Cancer categorized these dyes as group 3 carcinogens, and it banned Sudan dyes worldwide [16]. Although illegal, Sudan dyes are routinely detected in many types of food products, due to the fact that they make a spice look fresher, intensifying the color and making them appear of better quality, which translates into a higher price. Older spices may be added to freshly ground ones to increase weight [17,18]. Another illegal dye that is added illegally to food is rhodamine B, which has been detected in spice powder, spice soups, chili sauces, and colorful sweets. Usage of this dye generates a risk to the health of the consumer because it is potentially carcinogenic, neurotoxic, and genotoxic [19]. Apart from colorants, spices may be adulterated by addition of foreign matter with similar properties. The most frequently used adulterants are starch (e.g., maize, wheat, potato and rice) in turmeric, chili and curry powder, brick dust, sand, saw dust, and stone powder in chili powder, chalk powder in turmeric spices and dung powder, and common salt in coriander powder [10,20,21]. Moreover, tomato skin and brick powder were used for adulteration of paprika powder, while papaya seeds are convenient for adulterating pepper [10,22]. There are also reported cases of adulteration by the addition of inferior production—such as mixing fresh spices with spices that have had their valuable components removed (e.g., defatted paprika). The most difficult, from the identification point of view, are cases when the adulterant and the spice are parts of the same plant such as stamens of saffron in saffron threads or powder to increase their mass [23,24]. Among the various types of adulteration, mislabeling is a permanent problem for consumers in the world. For example, mislabeling of turmeric (*Curcuma longa*) as “Indian saffron” or “American saffron” causes consumers to buy products that they do not expect [25].

According to the annual report of the RASFF and Administrative Assistance and Cooperation (AAC) network, in 2019 more than 1500 notifications about food violation were reported. The notifications include 1433 incidents of mislabeling, followed by 375 incidents of unapproved treatment and/or process, and 210 incidents of replacement/dilution/addition/removal in product. The last position was held by non-compliance documents. In comparison to 2018, the number of alert notifications, implying a serious health risk of a product circulating on the market rose by 5% [26].

In this context monitoring food, spices and herbs authenticity which include checking their safety, quality, and compliance with its label description is an important aspect of international commerce today. For that reason, there is a compelling need for rapid high throughput validated analytical techniques detecting and preventing economically motivated fraud. The most popular techniques for this purpose are liquid and gas chromatography coupled with mass spectrometry (HPLC/MS and GC/MS), isotope ratio and elemental analysis, spectroscopic methods such as NMR, Raman, NIR and FT-IR which are nowadays combined with chemometric methods (Table 1). Among these methods, NMR spectroscopy plays a major role and is one of the most commonly employed techniques in the structural elucidation of organic compounds, in pharmacy, cosmetic and food control. Authentic products usually can be distinguished from counterfeits by a careful analysis of their chemical composition. On the basis of an ^1^H NMR spectrum, a wrong ingredient can be easily recognized and its structure elucidated in the case of an unknown compound and/or impurity. NMR has distinct advantages over other spectroscopic methods. One of the greatest advantages of NMR is its non-destructive and non-invasive nature. It is environmental friendly, relatively rapid and easy to use on a regular basis with minimum sample preparation. It provides a lot of information within a short analysis time, especially quantitative and structural information for components of complex mixtures without pre-isolation. Moreover, the data can be stored and reanalyzed. However, certain disadvantages have also been found such as some difficulties connected with overlapping of signals in multicomponent mixtures and sometimes the information for major compounds is enhanced, while that of minor components is masked. The problem of overlapping signals can be overcome by use of 2D NMR techniques or curve-fitting algorithms implemented in some standard NMR software packages (e.g., TopSpin, Bruker Biospin). In order to acquire a good quality spectrum, the laboratory must be equipped with a modern spectrometer ensuring high sensitivity and resolution. Furthermore, the NMR technique requires a highly specialized operator. Additionally, NMR is more expensive in comparison to other spectroscopic techniques, but in a single run the determination of various chemical compounds can be achieved [5,9,27,28].

The purpose of this article is to provide an overview of the application of NMR spectroscopy combined with chemometric methods in spices authentication and their quality control. Our attention was focused on different NMR approaches and their applications.

## 2. Chemometrics

The large amounts of analytical data obtained from the spectroscopic methods require more advanced calculations such as the chemometric approach [5,10,26]. By definition chemometrics is the chemical discipline which uses mathematical, statistical, and other methods to extract maximum relevant chemical information and to predict chemical data as well as to design optimal procedures and experiments. In the case of multicomponent mixtures, where one sample is usually characterized by minimum hundreds of signals, the interpretation and data exploration are very complex. Chemometric methods reduce the multidimensionality of data and allow discovery of the possible relations and/or existing differences between different groups of samples such as authentic and adulterated. Four different fields in which chemometric can be applied including:

- Data reduction—where complex and massive data are simplified;
- Classifying objects—by means of unsupervised (exploratory) methods or by means of supervised (discrimination) analyses;
- Predicting analytical parameters i.e., calibration methods;
- Data fusion.

However, before appropriate chemometric analysis, the raw NMR data requires some kind of preprocessing to minimize any undesired variability caused by changes of the line shape and chemical shift, noise, baseline shift, signal referencing, and signal intensity. Thus, the main goal of data preprocessing is to improve the quality of multivariate data in order to ensure reliable analysis. This aim can be achieved using mathematical transformations on the raw data. Defernez and Coloquhoun pointed out how an inconsistency of the peak position and resolution may affect chemometric analysis and how important the appropriate preprocessing of raw data is [29]. The influence of the baseline components can be eliminated by using polynomial baseline correction. To decrease the changes in chemical shifts spectra are divided into equal size segments named also bins or buckets. The size of buckets usually ranges from 0.01 to 0.05 ppm and can be performed by using Amix program for example (Bruker Biospin, Rheinstetten, Germany). To minimize the imperfection caused by differences in the pulse length, relaxation times, and dilution of samples the normalization procedures must be applied. The normalization relies on the elimination of systematic bias by scaling the signal elements to a constant factor. In practice, the spectral intensities for each sample are scaled to a constant, which may derive from an internal standard or from the sum of the intensities in the spectrum [30,31]. As a consequence of normalization, the data derived from all samples can be directly compared with each other. Apart from bucketing and normalization also scaling transformation must be performed. Scaling operation modifies the variance of variables in order to reduce the impact of molecules with higher concentrations. In complex mixtures the concentrations of individual components may be different, thus dominated ingredients caused larger variance than signals of components with lower concentrations, thus there is a need to scale the raw data. To eliminate the variance caused by different concentrations of the mixture components and to ensure the same influence to the results the auto-scaling operation should be performed. Prior to auto-scaling the mean-centering is applied, in which mean intensity for each of the variables is subtracted from each spectrum. The auto-scaling relies on dividing the entries in the mean-centered matrix by standard deviation. Apart from auto-scaling, the Pareto-scaling is also used, where each variable is divided by the square root of the standard deviation. All scaling procedures aim at ensuring that all components of the analysis are of equal importance. Detailed information about scaling and normalization procedures are described by Craig et al. [31].

The appropriately prepared data can be used for statistical analysis to extract the relevant information about the relations between the samples or samples and variables indicating which are similar, which are different or which are typical outliers. There are two main data reduction methods, which are routinely used for raw analytical data: principal component analysis (PCA) and partial least squares discriminant analysis (PLS-DA). The input information for these methods is a bucket table constructed from the spectra, where the rows refer to a sample (spectrum) and the columns to a variable (bucket). The resulting table may have an enormous size with hundreds or thousands of rows and columns, and variables may be correlated or not. By means of PCA the behavior of spectra can be checked in order to evaluate if all spectra behave similarly or differently. PCA is the most commonly used unsupervised method, which leads to reduction of the dimensionality of a data set consisting of many variables to a few, orthogonal principal components (PCs) describing maximum variations within the data set. The new PCs are the linear combinations of the original variables. The first principal component explains most of the variance present in the original data, while other PCs are characterized by decreasing share of variance. In most cases the first three PCs are used for analysis, and by applying this method the score and the loading plots are obtained. The score plot allows for observation of any groupings in the data set (e.g., if all spectra have similar position with respect to the corresponding part of variance), while the loading plot shows the relation between the variables in the new principal component space and original spectroscopic space. Another exploratory technique which provides information about the correlation structures among samples and/or variables is hierarchical clustering techniques (HCA). For the data analysis a dendrogram (tree) is used, which is hierarchical—large clusters are split into smaller ones. The most similar samples/variables are placed at the bottom branches of the dendrogram, while longer branches show an increasing dissimilarity within the data. The main disadvantage of the HCA method is that it does not work with huge data. Among the clustering methods not only hierarchical but also non-hierarchical and density-based methods can be distinguished. More information about clustering methods can be found in the reference [32].

For the detailed analysis and categorization of the samples supervised methods, which are controlled by human experts, such as PLS, a combination of PLS with discriminant analysis PLS-DA, orthogonal PLS (OPLS), linear discriminant analysis (LDA) or soft independent modeling of class analogy (SIMCA) should be used. These approaches aim to construct the rules, which will be helpful to distinguish two groups on the basis of differences found at the level of their chemical composition. In principle, supervised methods generate models to classify samples or predict information for a new unknown sample. By applying these methods samples can be assigned to an appropriate category connected with their geographical origin, originality or adulteration and concentrations values of compounds. The samples can be ordered to only one group (in the case of discriminant methods) or to one, more than one or any group (classification method). In PLS approach, two tables are used: X table, which contains the independent variables (e.g., NMR spectra), and Y table, which contains dependent variables (e.g., other spectroscopic data, any other sort of data such as concentration, origin etc.) usually encoded by ones and zeros. Contrary to PCA, which detects the direction of maximum variance in the X table, PLS tries to find the best correlation between the X and Y tables by means of relevant linear combinations of variables in both tables. The unknown samples are then assigned to the classes according to prediction of the PLS model. For example, the spectra with missing Y table information are tested and the model is used to predict it. PLS as well as the principal component regression (PCR) are also used for creation of calibration models [10,32].

The well-known pattern recognition method and supervised version of PCA is SIMCA, which allows to distinguish each class separately in principal component space. In this method, the comparison of the standard residual deviation of the sample with the mean standard residual deviation of a given class is performed and on this basis the information on the degree of similarity of the sample to the class is provided [10].

The major challenge for chemometrics is data fusion. In this approach a large amount of data from different techniques is integrated to achieve a more efficient and accurate model than that provided by the use of an individual technique. Low, mid, and high level of data fusion can be distinguished. For multivariate analysis in low-level data fusion as an input information the raw data from different techniques are taken to produce new raw data, while in the case of mid-level the most important variables extracted from each technique are used. As a consequence, the main disadvantage of these strategies is the higher calculation time. The high-level data fusion uses the models provided by each technique, thus the calculation time is reduced. Apart from data fusion approach also big data technology is a promising tool for the analysis of complex structured and unstructured data from various sources and origins [10]. The big data approach transforms raw data comprised of a heterogeneous mix of formats into a coherent usable resource suitable for analysis [33]. Further development of multivariate analysis methods seems to be inevitable due to more and more advanced instrumental techniques including also combined techniques.

Prior to chemometric approaches, spectroscopic analysis must be performed, thus the next chapter covers NMR methods in spices authentication.

## 3. NMR Techniques Used for Determination of Authenticity of Spices

The aim of this chapter is to present several examples of the use of NMR as the first line analytical technique in spices quality and authentication. In the last two decades, NMR spectroscopy has been successfully introduced in the food industry for the determination of spices authenticity, quality and geographical traceability. There are several NMR approaches which allow the achievement of these goals such as (1) conventional NMR (standard 1D and 2D NMR spectra), (2) quantitative NMR (qNMR), (3) high resolution magic angle spinning (HR MAS NMR), (4) NMR-based metabolomics, (5) diffusion ordered spectroscopy (DOSY), and (6) isotope ratio measured by NMR (SNIF-NMR, site-specific natural isotope fractionation). All these NMR techniques provide structural and/or quantitative information on the various components of complex mixtures as well as information on their origin and adulteration.

### 3.1. Conventional NMR

Standard one- and two-dimensional (1D and 2D) NMR spectra, in particular ^1^H NMR, are successfully applied for identification, authentication, fingerprinting and quality assessment. A large number of information about samples and their composition within a single experiment were obtained. Moreover, the experimental procedures such as preparation of the sample are relatively simple. Depending on the nature of the sample (solid, liquid) and type of analysis, different ways of preparing spice samples for analysis are required. Some samples require little or no preparation for NMR analysis. For the non-targeted (or fingerprinting) approach, preparation of the sample relies on dissolving the appropriate amount/volume of the sample in deuterium solvent, while in the case of targeted analysis an extraction or fractionation step is required. The most frequently used solvents in spices studied are D_2_O, MeOD-d4, DMSO-d6, and CDCl_3_. In the case of extraction, the selection of solvent depends on the compounds of interest. Moreover, the disturbances in the chemical shifts in the NMR spectra can occur due to the differences in pH of food extracts. This problem has been solved by using phosphate buffers in D_2_O as a solvent. In most cases, the mixture of MeOD-d4 and KH_2_PO4 buffer in D_2_O is used for the extraction of amino acids, carbohydrates, fatty acids, phenolic, terpenoids, and organic acids [34]. In order to obtain better sensitivity and resolution, liquid samples are used because non-liquid systems are characterized by strong anisotropic interactions that provoke line-broadening [35,36,37].

Sensitivity and resolution are the main parameters, which determine the quality of NMR spectra. The enhancement of the sensitivity may be achieved by using a higher magnetic field with improved electronics, cryogenically cooled probes, and small volume NMR tubes. However, good quality spectral resolution is the major challenge for NMR analysis of complex mixtures. As mentioned earlier, overlapping of signals is one of the main problems in the qualitative and quantitative analysis. Lack of signal separation results in errors in integration and makes identification and quantification impossible. Several methods are proposed to overcome this problem, such as multidimensional NMR or hyphenation NMR, where separation and isolation of the individual components using HPLC or GC prior to elucidation of their structures by means of NMR is required. However, both methods are not without drawbacks, because 2D NMR cannot resolve signals in some cases, while HPLC and GC are time-consuming, require expensive columns and consume much more solvents. An alternative method which provides a solution for the problem of overlapped signals was proposed by Monakhova et al. [26]. Independent component analysis (ICA) was applied for the resolution of NMR spectra of complex mixtures such as honey, soft drinks and liquids used in electronic cigarettes. The proposed method allows to obtain good quality spectral resolution of up to eight-component mixtures with correlation coefficients between resolved and experimental spectra not less than 0.9. By means of ICA strategy the relative errors in the recovered concentrations were below 12% [26,38].

To enhance the spectral resolution, particularly for ^1^H NMR, where signal splitting significantly reduces the resolution, and frequently leads to signal overlapping, the pure shift experiment was proposed. This method is also known as the broadband homodecoupling technique and relies on the suppression of the effects of homonuclear coupling interactions. The presence of homonuclear (e.g., ^1^H-^1^H) and hetronuclear (e.g., ^19^F-^1^H, ^31^P-^1^H) couplings leads to complex multiplet structures, which makes the analysis more difficult, especially in highly overlapped proton spectra as found in natural products, foods, and biomolecules. The conversion of all signals into singlets allows the problem of overlapping to be overcome and significantly boosts the resolution. To remove homonuclear scalar couplings different methods are used and most of them are described in the references [38,39]. Among all methods leading to fully-decoupled proton spectra, the pure shift yielded by chrip excitation (PSYCHE) is one of the easiest to use and offers the ultra-high resolution. Moreover, this method shows better sensitivity than most other techniques with data chunking and handles strong coupling. Pure shift techniques may be extended to multidimensional methods in order to make these spectra much easier to interpret (e.g., PSYCHE-iDOSY, PSYCHE-TOCSY etc.) [39].

Many studies have been carried out on different spices by means of ^1^H NMR but also two-dimensional spectroscopy including ^1^H-^1^H COSY, ^1^H-^1^H NOESY, ^1^H-^1^H TOCSY, ^1^H-^13^C HSQC, and ^1^H-^13^C HMBC, which are helpful in unambiguous identification of metabolites in complex mixtures. Apart from correlation, spectroscopy also resolved techniques such as 2D ^1^H-^1^H *J*-resolved spectra or diffusion ordered spectroscopy (DOSY) are applied, in particular in NMR-based metabolomic studies. *J*-Resolved spectra provided additional information about the coupling constant, while in the DOSY spectrum signals are resolved based on the molecular diffusion coefficient. Moreover, numerous combinations of 2D NMR experiments can be applied such as HSQC-TOCSY, DOSY-HMQC, DOSY-COSY, DOSY-NOESY which allow to collect two different 2D spectra simultaneously in a single experiment [40,41].

One of the main advantages of using NMR in spices analysis is repeatability and reproducibility of measurements over the long term. The quantitative comparison between sets of spectra recorded at different times can be achieved by normalization of all spectra to the reference peak derived from the internal standard added to all samples [35]. Moreover, NMR is one of the spectroscopic methods, which offers the unique opportunity to generate statistically equivalent signals and gives the possibility to compare spectra originating from a single sample recorded by different spectrometers. Gallo et al. conducted the study and developed the procedures to generate statistically equivalent signals from a number of different spectrometers for complex authentic wheat and flour matrices [42]. The authors organized an inter-laboratory comparison involving 36 NMR instruments and investigated which aspects affected the signal equivalence. Spectrometers were characterized by different hardware configuration, manufacturer, magnetic field (400–700 MHz), age etc. All participants received five NMR tubes prepared by using the same batch of the deuterated solvent and recorded a ^1^H NOESY NMR experiment with presaturation to remove the residual signal of water. In order to evaluate the precision five repetition of the experiment was conducted. This study showed that the satisfactory results for assessing equivalence provide the values of coefficient of variation (CV%) lower than 5%. The obtained results indicated that the value of CV% depends on the operator skills, while the software types or effect of the magnetic field strength has a very small influence. The highest values of CV% were obtained for spectra processed by 36 different operators, while the lowest CV% values were obtained when all spectra were elaborated by a single operator [42].

#### Applications

Lee et al. discriminated the geographical origin of Asian red pepper powders distributed in Korea on the basis of ^1^H NMR combined with multivariate analysis. Red pepper (*Capsicum annuum* L.) is one of the most popular vegetable in Asia commonly used as a spice in a powder form. It is obtained by grinding sun- or heat-dried peppers, however, consumption of this spice in Korea is higher than production, which in 2017 reached only 68,221 tons. Most of the demand for red pepper is covered by import from China and other Asian countries (226,776 tons). Due to the fact that Korean consumers prefer domestic red pepper powder, it is more expensive than that from import, thus mislabeling is one of the most frequently occurring type of fraud. NMR analysis were conducted for 62 samples of Asian red pepper powder including: 36 samples from Korea, 17 samples from China, and 9 samples from Vietnam. Moreover, 16 samples of red pepper powders were purchased from Korean markets, in which 8 were from Korea, 5 from China, and 3 from Vietnam. These samples were used as a blind sample to check if they were correctly classified according to their geographical origin. Samples for the measurements were prepared by sonication of 500 mg of red pepper powder in the mixture of methanol-d_4_ (1200 μL) and D_2_O (400 μL) with 1 *w*/*w*% DSS as an internal standard. After that samples were centrifuged and filtered (a pore size—0.45 μm) and next placed in a 5 mm NMR tube. All measurements were performed on Varian VNS 600 MHz spectrometer. Quantitative analysis was conducted for 14 well-resolved signals such as α-, and β-glucose, sucrose, linoleic acid, unsaturated fatty acid, alanine, tyrosine, phenylalanine, tryptophan, histidine, uridine (two peaks), adenosine, kaempferol. By means of ANOVA analysis significant differences between three geographical origins were found. ANOVA was performed on the 14 selected peak heights, among which seven peaks showed significant differences in the means of the three group. The results were summarized in the Table 2. The relative integration values of the seven selected metabolite peaks in ^1^H NMR spectra were used to perform canonical discriminant analysis, by means of which 15 of the 16 blind samples were classified according to their correct geographical origin. In one case Chinese spice was classified as Korean due to high levels of α- and β-glucose [43].

In a similar approach, Sobolev et al. investigated the chemical composition of red sweet pepper “Cornetto di Pontecorvo”. This *Capsicum annuum* ecotype is cultivated at Pontecorvo town in the Ciociara area of the Lazio region, and it is labelled as protected by designation of origin PDO, which requires open-air cultivation of peppers. This kind of pepper is characterized by an intensely sweet flavor and unique properties due to it being grown on soil of the volcanic nature. Recently, another cultivation strategy was proposed due to fungal infections of soil, which affect the growth and the yield of pepper. Greenhouse strategy allows the control of environmental parameters such as humidity, temperature, light intensity and the use of agrochemicals. The authors performed the study aimed at the morphological analysis, chemical composition and biological activity of extracts from red sweet pepper grown open field (OF) and greenhouse (GH). The peel, pulp and seeds of red sweet pepper (50 mg) were extracted with 50 mL of EtOH (70% *v*/*v*) for 15 days. After that the supernatant was filtered and evaporated. Two kinds of samples for pulp, peel and seeds were prepared for analysis: one by dissolving the dried organic fraction in the mixture of CDCl_3_/CD_3_OD (2:1) and the second by dissolving dried hydroalcoholic phase in D_2_O phosphate buffer with TSP. All spectra were acquired on a Bruker Avance 600.13 MHz. Apart from ^1^H also 2D experiments such as ^1^H-^1^H TOCSY, ^1^H-^13^C HSQC and ^1^H-^13^C HMBC were performed. Twenty-five different compounds in the hydroalcoholic extracts were identified, while in organic extracts an additional ten compounds were found. Some differences between peppers grown in OF and GH were found. The pulp and peel extracts from red sweet pepper cultivated in open-field was characterized by the higher concentration of valine, alanine, glutamine, asparagine, aspartic acid, GABA, and phenylalanine. In comparison to OF fruits, the red pepper grown in GH were featured by higher concentration of glucose. The main differences between chemical composition of the pulp and peel turned out to be higher concentration of glucose and fructose in pulp samples and the higher level of choline in peel was found. Furthermore, it was observed that the seed extracts consisted of the same compounds as peel and pulp, except for succinic acid, ascorbic acid and glycine. In the case of organic extracts there was observed that the molar ratio of linoleic/linolenic fatty acids in pulp equals 2.2 (for both OF and GH peppers), whereas in peel extracts it equals 1.8 and 1.4 for GH and OF, respectively. Moreover, peel extracts were characterized by higher content of carotenoid in comparison to pulp extracts. In the case of seed extracts, the lack of galactolipids, phospholipids, and carotenoids were observed [44].

In another study, conventional NMR was used by Al-Samydai et al. for the analysis of capsaicin from *Capsicum annum* grown in Jordan. This kind of pepper is one of the most popular in the world. Due to hot and spicy taste it is usually used in a good deal of cuisines, while owing to its anticancer, anti-inflammatory, antidiabetic, and anti-coagulant activity it is also demanded in the pharmaceutical industry. By Soxhlet extraction with *n*-hexane extract from *C. annuum*, collected from the Al-Gor region, was obtained. For purified capsaicin obtained by flash chromatography both ^1^H and ^13^C NMR experiments were conducted to identify its structure [45].

Black pepper (*Piper nigrum*) is known as king of spices due to the fact that it is the most commonly used spice in the world not only for cooking but also as a food preservative, an important agent in perfumery as well as an essential component in traditional medicine that helps in the treatment of flu, pain, chills, exhaustion fever, rheumatism and colds. Black pepper belongs to the family Piperaceae, and it is cultivated for its fruits, which are dried and used as a spice. The main bioactive constituent of black pepper is piperine (PPN), which is responsible for its pungent flavor, and plays an important pharmacotherapeutic role. For example, it is administrated to cancer patients undergoing radiotherapy due to its protective effects against radiation [46]. Ahmad et al. conducted studies aimed at evaluation of the quality and standardization of black pepper samples in terms of the content of the active component PPN, its toxicity, and biological activity. Three different geographical origins of black pepper (India, Pakistan, and Vietnam) were analyzed. Accelerated solvent extraction was applied for black pepper extraction. Samples for NMR measurements were prepared by vortexing of 10 mg of sample with DMSO-d6, followed by its sonification for 20 min. After that the solution was centrifuged and 600 μL of supernatant was transferred into a NMR tube. Both ^1^H and ^13^C NMR experiments were acquired using Bruker Biospin spectrometer operating at proton frequency 300 MHz. All black pepper samples derived from different origins were characterized by the same signals for example from piperidine ring, which confirmed the presence of PPN, amide and pyrrolidine derivatives. For quantification of PPN the UHPLC-DAD was applied. The concentration of PPN decreased in order from Vietnamese, Indian to Pakistani samples. The higher amounts of PPN found in the Vietnamese black pepper resulted in its higher antioxidant and cytotoxic activity. Moreover, the Vietnamese black pepper was characterized by large content of macronutrients and micronutrients. The obtained results suggested that Vietnamese samples have better quality in comparison with the other two samples [47].

In another study Verma et al. proposed various 2D NMR methods for the identification of compounds present in coriander (*Coriandrum sativum* L.) The utility of pure-shift proton spectrum along the indirect F_1_ dimension (F_1_-PSYCHE-TOCSY) as well as *J*-resolved NMR, TOCSY and HSQC for the analysis of complex mixture was checked. Coriander—called also Chinese parsley—belongs to the family Apiaceae and has its origins in Southern Europe, North Africa, and southwestern Asia. It is cultivated for its fruits, which are used as a spice or for essential oil production. The coriander fruits are also known for their health benefits such as antibacterial activity against *Salmonella choleraesuis*. Moreover, coriander has been used in the treatment of join pain in rheumatism and indigestion. The chemical composition of coriander seeds includes a wide variety of monoterpenoids, aromatic glycosides, and monoterpene glycoside sulfates, a wide range of essential oils, monoterpenes and fatty acids. After extraction, the dried powder was mixed with D_2_O and phosphate buffer in D_2_O, and vortexed. After that, the mixture was centrifuged and supernatant was transferred to a NMR tube, into which a coaxially inserted tube with TSP (0.5 mM) was also placed. All spectra were acquired on a Bruker Avance 800 MHz equipped with ^1^H/^13^C/^15^N CPTCI cryoprobe. The authors demonstrated the advantage of ultrahigh resolution spectroscopy, which allowed the identification and unambiguous assignment of 36 polar metabolites without prior separation by using chromatographic methods. By the application of the ultrahigh resolution of F_1_-PSYCHE-TOCSY, nine compounds in crowded sugar region were assigned, while their identification was not possible in overlapped TOCSY spectrum. Moreover, it was found that better assignment of compounds was possible to be achieved when F_1_-PSYCHE-TOCSY was recorded with the same acquisition time as was for regular TOSCY [48].

Moreover, conventional NMR approach was applied for structure elucidation of bioactive compounds isolated from spice *Aframomum melegueta*, commonly known as Guinea pepper, widely cultivated in the tropical regions of Africa. Due to numerous pro-health properties, this spice is used for treatment of stomach ache, measles, inflammatory conditions, hemorrhage etc. On the basis of 1D and 2D NMR spectra, ten constituents isolated from this plant were characterized, of which two were undescribed compounds [49]. In similar approach Karakaya et al. elucidated structures of bioactive compounds isolated from different *Ferulago* species cultivated in Turkey. *Ferulago* species are employed as spice, food for goats and deers as well as remedy against headache, snake bites, ulcers, spleen diseases. Fourteen compounds were isolated and then characterized by means of NMR and MS approaches [50].

### 3.2. Quantitative NMR (qNMR)

NMR has the unique property of being intrinsically quantitative due to the fact that the integrated intensity of a resonance signal is directly proportional to the number of resonant nuclei by that signal under appropriate recording and processing conditions. To obtain high-quality and reproducible NMR spectra in order to maximize accuracy, a number of parameters need to be carefully checked from sample preparation to data recording and processing. First, quantitative analysis by means of NMR requires a reference compound for calculating the concentration of an analyte. It can be an internal, external, or electronic standard. The ideal reference should not have overlapping signal with other components of the mixture, the relaxation times T_1_ of its protons should be as short as possible, should be available in a highly purified form, long-term stable, non-volatile, non-hygroscopic, soluble in most of the NMR solvents, and of defined concentration [35,51,52]. In the case of an internal standard, a measured amount of a reference compound such as tetramethylsilane (TMS, organic solubility) or sodium 3-(trimethylsilyl)-propionate-2,2,3,3-d4 (TSP, aqueous solubility) or 3-(trimethylsilyl)-1-propane sulfonic acid sodium salt (DSS) for aqueous solutions is added to the sample. Due to the high volatility of TMS it is recommended to use other reference for qNMR such as maleic acid, formic acid, 1,3,5-trioxane, or *p*-toluenosulfonic acid. Moreover, in the case of TSP and DSS, which are known to bind proteins and fatty acids, it is important to choose another reference not affecting the quantitative accuracy. The external standard approach eliminates contamination of a precious analyte sample by the reference, because the standard is placed in a coaxial stem insert. In addition, there is a need to calibrate the volume of analyte solution and reference solution, which should be equal. In order to avoid problems with shimming the internal standard and the analyte should be dissolved in the same solvent. After recording the spectrum the capillary is removed, rinsed and can be reused for quantitative analysis of other samples. Apart from an internal and external references the ERETIC approach (electronic reference to access in vivo concentration) is used in qNMR by placing an electronic signal in a free space of a sample spectrum. This method relies on the generation by the second channel of the probe a pseudo-FID, which is characterized by similar parameters such as the real NMR signal (e.g., frequency, magnitude and phase). Prior to qNMR measurements the ERETIC signal should be calibrated by means of solution with known concentration, and after calibration the concentration of any other compound can be quantified [35,51].

Prior to quantitative analysis, several experimental parameters should be optimized to ensure accuracy and precision, such as pulse excitation, relaxation delay, acquisition time etc. (Table 3). The most frequently used pulse angle in qNMR is 90°, which normally provides maximum intensity. Furthermore, sufficiently long repetition time must be matched to allow the equilibrium magnetization state to be re-established before the beginning of the next pulse cycle. In practice, it should be at least seven times longer than longitudinal relaxation time (T_1_) to measure 99.9% of the equilibrium magnetization. The precise quantitative results can be also achieved when signal to noise ratio (S/N) equals 150:1 at least. In that case the integration error can be maintained within 1% [35,51,53]. S/N can be influenced by the number of scans. Thus, if the concentration of the mixture is very low, the number of scans can be increased to achieve an appropriate S/N. Moreover, an adequate digital resolution (at least five data points above the half-height) and acquisition time (about three times longer than transverse relaxation time T_2_) must be ensured. For typical ^1^H NMR spectrum 32,768 data points are required for a digital resolution of 0.15 Hz (at 400 MHz spectrometer) [35,51]. It is worth remembering that better digital resolution is connected with better peak definition and more precise integration. To avoid problems with the presence of spinning sidebands (which are associated with the rotation of the NMR tube) overlapping with analyte signals, in quantitative measurements spinning of the sample is not used. However, in the case of interferences caused by the presence of ^13^C satellites, which leads to distortion in intensity (due to the nuclear Overhauser effect (NOE)), the heteronuclear decoupling must be performed. The NOE is caused by dipole–dipole coupling interactions, which are through space and are distinct from scalar coupling contributions. As a consequence the change in the signal intensity is observed. In the case of ^13^C or other heteronuclei the increase in the signal intensity due to broad-band proton decoupling (or in the case of ^1^H experiments with broad-band decoupling of X nuclei) is caused by the perturbation of the equilibrium population of protons because of decoupling pulses, which in turn affects the population and thus the signal intensities of the neighbouring ^13^C nuclei that are dipolar coupled to protons. In the case of nuclei such as ^15^N with negative gyromagnetic ratios, the NOE is negative and there is reduction in signal intensity [30,51,53].

Not only acquisition parameters should be carefully checked to obtain accurate quantitative results, but also processing parameters such as phase correction, baseline correction, deconvolution, and integration. Both improper phase correction and incorrect baseline introduces significant errors in absolute or relative concentrations of compounds. In the case of phase correction, the manual correction is preferred over automatic due to the fact that signals with low concentration get distorted during automatic phase correction. The most important step in qNMR analysis is integration of peak area. In order to reduce the operator error it is recommended to perform integration from 5 to 10 times and to take an average of all values. Moreover, in the case of overlapping signals the deconvolution is required to determine the contribution of an individual peak to the total area [51,53].

#### Applications

Farag et al. [54] proposed a quantitative NMR approach to discriminate two cinnamon species of different geographical origin, *Cinnamomum verum* and *Cinnamomum cassia*. Cinnamon is one of the most commonly used spices in the world. It is made from the bark of species of the cinnamon tree growing in Southeast Asia, China and Australia. Among varieties of cinnamon these two species are the most famous. *Cinnamomum verum* is also known as a true or Ceylon cinnamon in reference to its origins, and it is characterized by delicate flavor, golden color, many thin layers of bark and healthy benefits such as antifungal, anti-allergy, anti-diabetic activity. It is also used in blood circulation disorders and inflammatory disease. Due to its health benefits Ceylon cinnamon is more expensive than *C. cassia*. In comparison to Ceylon cinnamon, the Cassia cinnamon (also known as Chinese cinnamon) has a red-brown color, is rolled in thick sheets, has an intense flavor and contains higher concentration of coumarin. With regard to the coumarin content the regular consumption of Chinese cinnamon at high amounts may lead to liver failure. Prior to NMR analysis cinnamon samples were extracted, then dried extracts were suspended in methanol-d4 using hexamethyldisiloxane (HMDS) as an internal standard. In the next step samples were centrifuged and supernatant was placed in a 5 mm NMR tube. All spectra were performed on an Agilent VNMRS 600 spectrometer operating at a proton frequency 599.83 MHz. Experiment conditions were selected in accordance with requirements for quantitative NMR analysis. Samples of Ceylon and Chinese cinnamon originating from Brazil, Jordan, Romania, Egypt and Tanzania were analyzed. The major flavor compounds detected in cinnamon samples included glycerol, acetic acid, (*E*)-cinnamaldehyde, (*E*)-cinnamaldehyde dimethyl acetal, *o*-hydroxycinnamaldehyde, (*E*) and (*Z*) cinnamic acid, and eugenol. PCA and OPLS-DA analyses were performed in order to differentiate both cinnamon species. It was found that the presence of eugenol may serve as a biomarker for authentication of Ceylon cinnamon extracts as it was detected in almost all *C. verum* samples except for *C. verum* from Jordan, and it was present in only one sample of *C. cassia* from Tanzania. Moreover, the fatty acids can be used as an indicator of the presence of Chinese cinnamon, owing to their higher concentrations in *C. cassia* extracts.

Quantitative approach was also applied in papers describing NMR-based metabolomics and HR MAS NMR approaches [43,55,56,57,58].

### 3.3. HR MAS NMR High Resolution Magic Angle Spinning

To study semi-solid and gel-like samples a high resolution magic angle spinning technique can be applied. This technique was successfully introduced to study the metabolic profile of biological samples, but also it can be easily applied for the study of metabolic profile and their changes in food samples. In the case of solid samples, the main problem is the strong dipolar and quadrupolar interactions as well as chemical shift anisotropy and magnetic susceptibility, which provoke line broadening effects. Both solid- and liquid-state advantages are combined in HR MAS methods. Contrary to liquid samples, where NMR tubes are used, solid materials are placed in the zirconium rotors (e.g., 50 μL, 4 mm) with a small amount of deuterated solvent to ensure both the molecular spin mobility and the deuterium lock. By spinning the sample during acquisition around an axis inclined 54.7° (the so-called magic angle) to the direction of the static magnetic field, the dipolar couplings are reduced. The dependence of anisotropic nuclear interaction on the angular relationship (3cos^2^β−1), which equals zero when β is 54.7, was described by Andrew and Newing in 1958 [59]. In consequence of spinning the sample at magic angle all broadening factors are averaged and thus the signal resolution achieved by means of HR MAS technique is comparable to those performed in liquid-state. The spinning rate must be at least of the same order of magnitude as the anisotropic spin interaction or much larger. Moreover, the spinning rates are usually chosen to ensure that the spinning sidebands appear outside of the spectral region of interest. However, depending on the sample and its stability, spin rates from 3 to 6 kHz are recommended. Spinning around the magic angle splits the broad signals into narrow lines, but in the case of large molecules the broad signals are still obtained in the HR MAS spectrum. In order to reduce the broad resonances caused by macromolecules with short transversal relaxation times, T_2_ the spin-echo sequence with long echo-times before acquisition should be applied. Due to the elimination of signals from macromolecules, the signals from low molecular weight compounds may be observed [37,60].

HR MAS NMR has many advantages. For example, it is a rapid and non-destructive method, and offers a unique possibility of measuring samples without any chemical and physical preparation. Moreover, qualitative and quantitative analysis can be conducted and obtained spectra are characterized by highly resolved peaks.

#### Applications

The HR MAS NMR technique has been successfully applied for assessing the metabolome of food products. The metabolic changes and chemical composition of typical products of the Mediterranean diet (such as Protected Geographical Indication (PGI) cherry tomato of Pachino, the PGI Interdonato lemon of Messina, Protected Designation of Origin (PDO) extra virgin olive oil of Sicily, the mozzarella cheese from Campania buffalo milk) were determined by means of this technique [61]. Moreover, HR MAS NMR was applied to study quality and fish freshness as well as wheat products and bread [37]. The HR MAS technique was also employed to spice analysis such as Italian sweet pepper and the Traditional Italian Food Product (PAT) red garlic of Nubia [57,62].

The metabolic profile of the PAT red garlic of Nubia and the quantification of its main metabolites was determined by Ritota et al. Garlic is one of the most widely used spices in many parts of the world due to its unique flavor and taste as well as their health benefits such as antimicrobial, anticancer, antioxidant activities. The authors conducted a comprehensive chemical characterization of the red and white garlic cropped in different Italian regions. Moreover, the provenance of a total of 48 garlic samples collected during the period 2009 and 2010 was studied. Thirty-six red garlic samples (12 harvested in Sulmona, 12 in Casterlliri and 12 in Proceno) and twelve white garlic samples collected in Piacenza were analyzed by means of HR MAS. Samples for the analysis were obtained by placing about 5 mg of garlic in a rotor (4 mm, 50 μL) and a volume of 40 μL of D_2_O phosphate buffer with 0.5% TSP was added to each sample. All spectra were recorded on a Bruker Avance spectrometer operating at a ^1^H frequency of 400.13 MHz. The ^1^H HRMAS NMR spectrum of garlic showed signals belonging to carbohydrates (e.g., α- and β-glucose, sucrose), organic acids (e.g., citric, malic, formic and fumaric acids), amino acids, fatty acids (e.g., linoleic and palmitic acids), organosulphur compounds (e.g., allicin, S-allyl cysteine) and other compounds (e.g., choline, ethanol, methanol, trigonelline pyrazine and stherols). Allicin and S-allyl cysteine were the main compounds responsible for antimicrobial and antifungal properties of garlic, respectively. It was found that the red garlic of Nubia was characterized by higher level of S-allyl cysteine, pyruvate, and riboflavin in comparison with other red garlics. The concentration of carbohydrates was comparable in both red garlic from Nubia and Sulmona. Moreover, the red garlic of Sulmona was characterized by the presence of diethylthiophosphonate, which is widely used as a pesticide. Analysis of data by means of PLS-DA allowed to discriminate the red from white garlic samples on the basis of formiate, citrate, and two unknown compounds (signed as 1 and 2). The red garlic from Proceno was discriminated on the basis of fatty acids, γ-aminobutyrate (GABA), lysine, tyrosine, tryptophan, phenylalanine, methanol, allicin, and the unknown metabolite 2, while red garlic of Castelliri was differentiated by means of palmitic and linoleic acids, alanine, lysine, arginine-GABA-lysine, allicin, and the unknown metabolites 6, 8 and 9. The differentiation of the red garlic of Sulmona was obtained on the basis of GABA, citrate, formiate, and the unknown compounds 1 and 2. By means of ^1^H HRMAS and multivariate analysis samples of red and white garlics were classified and their geographical origin were determined [57,61].

After garlic, onion is one of the most important culinary vegetable and is widely used for cooking as a spice or raw food. Similarly to garlic, leek, shallot and chives onion also belongs to *Allium* family plants, thus contains bioactive organosulphur components responsible for therapeutic effects and health benefits such as antimicrobial, anticancer, and antioxidant activities. It was found that regular consumption of both onion and garlic may protect against cardiovascular and neurological diseases as well as arteriosclerosis. Jo et al. have investigated the potential of ^1^H NMR and ^1^H HRMAS NMR in the identification and detection of the quantitative differences in metabolites with origins of growth and harvest. It is known that type and concentration of compounds may vary depending on the origin of products. Factors such as climates, humidity, temperature, sunshine etc. may affect the chemical composition of products, thus the authors studied garlic and onion from Korea and China to discriminate country of origin of agricultural products. For ^1^H NMR analysis 10 mg of powdered samples were dissolved on 600 μL phosphate buffer (D_2_O, 10 mM) with TSP, after that samples were sonicated and vortexed. A volume of 400 μL of final extract was placed in the 5 mm NMR tube. In the case of HR MAS studies about 2 mg of sample was placed into 4 mm HRMAS rotor, and 30 μL of 0.01 M D_2_O phosphate buffer was added. All experiments were performed on Bruker Avance III HD 400 MHz. Twenty garlic metabolites were assigned by means of ^1^H NMR and twenty-one using ^1^H HRMAS NMR, while in the case of onion twenty-four metabolites was detected by both techniques. Chinese garlic was characterized by the presence of peaks from tyrosine and tryptophan and the higher intensity of metabolites at the region from 1.0 to 1.5 ppm in comparison to Korean garlic. Moreover, HR MAS NMR studies indicated that Korean garlic samples were characterized by the presence of organosulphur component at about 5.2 and 5.8 ppm and lower intensity of fatty acids, amino acids and ethanol than Chinese. In turn, Chinese onion showed higher level of metabolites in a range from 1.0 to 3.0 ppm compared to Korean. PCA analysis was applied to discriminate onion and garlic origins. Distinction of garlic from onion was possible on the base of valine, alanine, aromatic compounds and ethanol. Moreover, PCA analysis allow to distinguish four groups: Korean garlic, Korean onion, Chinese garlic, and Chinese onion with confidence level of 95% [58].

Ritota et al. have also investigated the potential of ^1^H HR MAS NMR to assess the metabolic profile of sweet pepper (*Capsicum annuum* L.) growing in various parts of Italy. The study was conducted for two different cultivars “Corno” (101 samples) and “Cuneo” (152 samples). All samples were prepared in the same way: 25 mg of sweet pepper was inserted into HRMAS rotor (4 mm, 50 μL), and a volume of 25 μL of D_2_O phosphate buffer with 0.5% TSP was added to each sample. All spectra were acquired on Bruker Avance 400 MHz spectrometer. The chemical composition of sweet pepper was characterized by the presence of numerous amino acids (e.g., alanine, arginine, asparagine, GABA, glutamine, glutamate, leucine, isoleucine, phenylalanine, threonine, tyrosine, tryptophan, tyrosine, and valine), carbohydrates (e.g., α- and β-glucose, β-fructopyranose, sucrose, α- and β-fructofuranose), fatty acids (e.g., palmitic, stearic, oleic, linoleic and linolenic acids), organic acids (e.g., citric, ascorbic, malic, fumaric, formic acids) and other metabolites (e.g., cinnamic compounds, trigonelline, C4-substituted pyridine, choline, creatinine, and creatine). To discriminate samples of sweet pepper according to the variety and to the geographical origins, multivariate analysis was applied. By means of PLS-DA peppers were classified on the basis of their cultivars and the biochemical compounds responsible for this discrimination were identified. It was found that “Corno” peppers were characterized by the highest concentrations of sucrose, glucose and polyunsaturated fatty acids, while “Cuneo” peppers by the highest levels of glucose, arginine, GABA, acetate, and fatty acids. Thus sugars, organic and fatty acids proved to be the main biomarkers allowing to differentiate these two cultivars. Moreover, the attempt to define the geographical origin was made. In the case of cultivars “Corno” (101 samples of peppers), three different geographical areas were considered: Cuneo (83), Turin (10), and Asti (8). Pepper samples from Asti were differentiated on the basis of low levels of glucose, fructose, vitamin C, asparagine and higher concentration of glutamine and malic acid. Samples derived from Turin were characterized by high concentration of asparagine and ascorbate, and low content of malate, cinnamic acids, phenylalanine, and unknown compound at 5.9 ppm, while in peppers from Cuneo high concentration of sugars, malate, unknown compound (at 5.9 ppm), and low content of asparagine, malate, and vitamin C was found in comparison to peppers derived from Asti and Turin. The same analysis was conducted for cultivar “Cuneo” (32 samples from Sicily, and 121 from Piedmont within 99 were from Cuneo and 21 from Turin). Samples from Sicily were discriminated on the basis of low content of *cis* olefins, fructose, β-glucose, unsaturated fatty acids, and high levels of acetate, sucrose, glutamine, GABA, and arginine. Low amounts of sugars, and high level of malate and asparagine were found in pepper samples from Turin. In the case of pepper samples derived from Cuneo high concentrations of fructose, glutamine, and fatty acids were determined, while asparagine, malate, sucrose, and glucose were found at lower concentration [62].

In another study Hu et al. investigated the potential of ^1^H NMR, qNMR and ^1^H HRMAS NMR to detect the adulteration of paprika powder by Sudan dye I. As mentioned in the introduction, cancerogenic dyes such as Sudan I are added to spices for an apparent freshness and to reduce the cost. For validation of both NMR approaches, prior to analysis of purchased paprika powder samples were checked for the presence of Sudan dye by means of HPLC-DAD (high performance liquid chromatography-photodiode array detector). After that Sudan I stock solution in acetonitrile and in DMSO-d6 were prepared. An amount of 200 mg of paprika powder were spiked with 400 μL of Sudan I acetonitrile solutions at various concentrations, then vortexed, sonicated and centrifuged. Supernatant was evaporated and re-dissolved in 500 μL DMSO-d6 and placed in 5 mm NMR tube. For determination of the linearity and repeatability paprika powder samples were prepared three times with Sudan I at 20, 50, 100, 250, and 500 mg/kg. The accuracy was estimated for paprika samples spiked with 50, 75, 125, 200, and 800 mg/kg. All experiments were performed on a Bruker spectrometer operating at proton frequency 600.13 MHz. In the case of HR MAS studies 10 mg of paprika powder were placed into standard 4 mm rotors and spiked with 45 μL Sudan I in 50% DMSO-d6 at various concentrations. Five samples of paprika powder with different amount of Sudan I (225, 675, 1350, 1800, and 800 mg/kg) were prepared for the linearity and repeatability determination, while the accuracy studies were conducted for samples with 337.5, 450, 1125, 1575, and 3150 mg/kg of dye. All spectra were recorded on Bruker 600.25 MHz spectrometer. Results showed that both ^1^H NMR and ^1^H HRMAS NMR were possible to be applied for rapid and accurate determination of Sudan dye addition to paprika powder samples. Analysis performed in liquid NMR were characterized by an average accuracy of 98% (range 94–104%) with the 6.7 mg/kg limit of detection (LOD), 22.5 mg/kg limit of quantification (LOQ), and 35 min the overall time of analysis. Moreover, the relative standard deviation (RSD) was estimated at an average of 4.6%. In comparison to ^1^H NMR, determination of Sudan I in paprika powder by means of HR MAS NMR was featured by an average of 3.9% RSD, an average of 105% accuracy, and 32 min overall time of analysis. The LOD and LOQ values are evaluated at 128.6 and 313.7 mg/kg, respectively [56].

Described examples indicate that HR MAS NMR technique is an excellent analytical tool, which allows to determine not only the chemical composition but also adulteration of spices and determination their geographical origins.

### 3.4. NMR-Based Metabolomics

Food metabolomics is a comprehensive qualitative and quantitative analysis of biochemical composition of food products including all low molecular weight components such as primary and secondary metabolites. Due to the change of our lifestyles and raising awareness on the influence of food on our health, foodomics has become one of the most studied research field. People think that unhealthy food including worse quality and adulterated spices may be responsible for different types of disease such as cancer, high blood pressure, intestinal problem, and allergy [20,63]. Food metabolomics includes not only the evaluation of the molecular composition of food, which is very complex because it consists of hundreds or even thousands of compounds, but also it is focused on the interactions between food ingredients and studies the impact of food storage, transportation, and processing on its composition. Several aspects such as identification of unknown samples, classifying various groups of samples as well as investigating the relationship between food composition and spectroscopic data are the main goals of these studies [30,34]. Research strategies in metabolomics can be divided into two distinctive but complementary approaches—targeted and non-targeted (other term: metabolic fingerprinting) analyses. The main goal of targeted approach is quantitative analysis of predefined group of biochemically characterized and interpreted metabolites. Prior to quantitative analysis, extraction techniques allow to isolate of specific markers are applied, and then identification, quantification and comparison with established limits according to validated and internationally agreed procedures are conducted [30,34,42]. On the other hand, the non-targeted approach relies on the comprehensive analysis of all measurable metabolites in a sample which allows for the detection of known and unknown compounds in the mixture. This kind of analysis is inclusive of chemical unknowns, and thus provides a fingerprint of the sample for discovery of novel metabolite species, pathways and targets. Among different technological platforms, two leading analytical approaches (NMR and MS) have been successfully applied for metabolic fingerprinting. Both techniques have their limitations and advantages, however NMR offers unique advantages over MS-based methods, because it is a universal detector for all molecules and allows to identify compounds that are difficult to ionize or require derivatization. In addition, NMR provides the possibility for identification of compounds with the same masses but with different isotopomer distribution [64].

The general procedure of sample preparation was described in Section 3.1, but in the case of metabolic approaches the appropriate preparation of sample is required to obtain reproducible and reliable results. In order to avoid the introduction of variance, all samples should be collected and prepared in an identical way and samples selected for the analysis should be representative for the total population. Moreover, in the case of samples which may undergo biochemical changes, freezing at −80 °C immediately after collecting the sample is required. Biochemical changes may also occur during extraction process, thus in the case of metabolomics studies drying, freeze-drying and keeping the sample at low temperatures is recommended. Furthermore, for samples which require grinding prior to analysis this process should be also carried out at low temperature or with addition of solvent that denatures enzymes involved in the metabolite alteration [34]. Apart from sample preparation, spectral acquisition and processing must also be identical for all samples to ensure reliable analysis.

#### Applications

NMR-based metabolomics was used by Saviano et al. for chemical fingerprinting of three edible Italian *Allium cepa* L. The chemical profile for white onion (WP, var. *San Pietro*) was determined for the first time and compare with metabolome from the red onion (RT, var. *Tropea, Rosa di Tropea*) and the yellow onion (CM, var. *Montoro, Ramata di Montoro*). Characteristics of plants from *Allium cepa* L. family was described in Section 3.3. Onion bulbs accumulate about 80% of carbohydrates, mainly glucose and fructose, but also sucrose, fructans and low molecular weight fructo-oligosaccharides, which may prevent against cold and drought condition. White *San Pietro* onion is produced in the Isernia area, and it is known for its sweetness and low pungent smell. NMR analyses were performed for the aqueous and chloroform extracts obtained from fresh onion and after 9-month storage at temperatures of 20–25 °C. The extraction process resulted in three solution phases. Polar metabolites were found in the aqueous phase at the top, while denatured proteins in the middle, and lipids in the chloroform phase at the bottom. Both the aqueous and lipid phase were vacuum dried and kept at −80 °C before analysis. Samples for measurements were prepared by dissolving in D_2_O phosphate buffer with TSP. All experiments were recorded on a Bruker Avance III 600 MHz spectrometer. Thirty metabolites such as aromatic carbohydrates, organic acids, amino acids and organosulfur compounds were identified on the basis of 1D and 2D experiments. The metabolic profile of white onion was characterized by carbohydrates (about 95%) and by the presence of fructo-oligosaccharides (FOS), which were not observed in red and yellow onions. In the case of amino acids content, it was found that the concentration of these compounds is the highest in yellow onion, it decreased in red onion, and was the lowest in white onion. In fresh white onion samples, low content of amino acids was observed. Furthermore, in the metabolic profile of white onion the lack of pyruvate and α-hydroxybutyrate as well as the low content of all sulfur component was observed. The highest level of sulfur components and amino acids were found in yellow onion samples. The total phenolic content, both in fresh and storage samples, decreased on going from red to white onion, while the total flavonoid content decreased in order from red to yellow onion samples. For stored white onion samples, higher level of sulfur compounds, and free mono and disaccharides was observed due to hydrolysis of the fructo-oligosaccharides, while in red and yellow onions the concentration of carbohydrates and sulfur compounds slightly decreased. To classify onion samples according to the metabolic profiles, expressed by the different cultivars, the PCA and PLS-DA analyses were applied. The results were summarized in the Table 4 [65].

In another study Becerra-Martinez et al. discriminated serrano peppers cultivated in two distinct regions based on ^1^H metabolic profile combined with chemometric approaches (PCA and OPLS-DA). Green chili pepper (*Capsicum annuum* L.) is one of the most popular spices in Mexico widely used for the preparation of meals as well as a natural analgesic. The metabolic profile for peppers cultivated in Veracruz and Oaxaca were determined. Prior to NMR analysis serrano pepper samples were squeezed in a mortar and after centrifugation of the juice, the upper phase was mixed with D_2_O with mM TSP, 10 mM EDTA and 2 mM NaN_3_. NMR analysis were performed for 600 μL of this solution placed into NMR tube. All ^1^H experiments were conducted on a Bruker 750 MHz spectrometer equipped with a 5 mm TXI cryoprobe. 2D experiments were carried out using a Varian NMR system operating at a proton frequency of 499.8 MHz. The chemical composition of the aqueous phase of the serrano peppers consisted of 40 metabolites. NMR spectra were dominated by signals from α- and β-glucose, fructose and sucrose. Moreover, numerous amino acids, carboxylic acids, alcohols as well as nucleosides were found. Based on PCA and OPLS-DA models, the statistically significant differences between two groups of chili peppers were demonstrated (Table 5). Given the fact that the differential organic acids were found in serrano peppers from different origin, it was demonstrated that the chemical composition depended on several factors connected with growing area such as temperature, sunshine, or humidity [66].

In another article Villa-Ruano et al. applied a similar protocol for the identification of the main metabolites of ten new races of *C. annuum* cv. serrano (labeled Camino real, Centauro, Chiser, Coloso, Diablo, Estrella, Feroz, HS 52, and Impala). Moreover, qNMR approach was applied to estimate the concentration of the main metabolites characterizing these races. All samples were prepared on the basis of protocol described by Becerra-Martinez et al. [66]. NMR experiments were performed on a Bruker 750 MHz spectrometer equipped with a 5 mm TXI cryoprobe. PCA and PLS-DA analyses were conducted in order to identify differences among the samples, while ANOVA was applied to determine significant differences in metabolite levels. The metabolic profile obtained by means of ^1^H NMR spectra consisted of 48 metabolites, including amino acids, organic acids, alcohols, sugars, and nucleoside. Based on PLS-DA model two defined groups were differentiated. The group A included races Diablo, Estrella and Impala, while to group B belonged remaining seven races. A higher level of citric acid and sucrose was detected in the group A, whereas higher concentration of fructose, fumaric acid, glucose, and malic acid was found in the group B. Low concentrations of citric acid were found in almost all races, however, the highest concentration of this metabolite (67.09 mM) was detected in the Estrella race, while two times smaller in Diablo race (29.71 mM) [54].

In a similar approach Florentino-Ramos et al. performed the studies focused on determination of the metabolomics fingerprinting of 11 chili peppers cultivars of *Capsicum annuum* species. To classify the *Capsicum annuum* cultivars regarding their metabolic profile, PCA and LDA analyses were applied. Moreover, the metabolomic differences and similarities among different cultivars was detected by means of PCA, while hierarchical cluster analysis HCA was used for the separation of the cultivars according to the phylogenetic trees. All samples were prepared in accordance with protocol reported by Becerra-Martinez et al. [66]. NMR experiments were performed on a Bruker 750 MHz spectrometer equipped with a 5 mm TXI cryoprobe. Based on ^1^H NMR spectra, 44 metabolites were detected such as sugars, amino acids, organic acids, polyphenolic acids, and alcohols. A total of 110 samples were processed in chemometric approach (10 replicates for each cultivar). A similar type and concentration of metabolites were found in cultivars árbol, serrano, cuarsmeño, and chorro, while a significant dissimilarity was detected for *C. annuum* cultivars such as anaheim, chorro, hungaro, poblano, árbol, chilaca, and caribe. Furthermore, other similarities were observed in cultivars Caribe, hungaro, poblano, anaheim, and chilaca. ^1^H NMR spectra of all cultivars were dominated by signals from amino acids, however, only in the case of samples from cultivars anaheim, caribe, chorro, hungaro. mirasol, and poblano, methionine was observed. Malic acid and fumaric acid served as biomarkers indicating cultivars hungaro and árbol, respectively. A little content of sucrose characterized cv. agua and cv. anaheim samples, while the lowest concentration of fructose and glucose was found in cv. chorro. In the case of cv. mirasol the highest content of galactose was detected, while cv. chilaca was characterized by higher level of ascorbic acid [67].

The chemical divergences among ten new species of japaleño peppers were assessed by means of NMR and machine learning algorithms and reported by Ramirez-Meraz et al. Aqueous extraction samples were prepared as describe by Becerra-Martinez et al. [66]. All NMR experiments were recorded on a Bruker 750 MHz spectrometer equipped with a 5 mm TXI cryoprobe. For statistical analysis PCA and OPLS-DA were used. To determine differences between metabolite levels, ANOVA were performed. The metabolic profile of *C. annuum* cultivars japaleño consisted of 48 metabolites including amino acids, carboxylic acids, alcohols, sugars, and nucleosides. The clear differences among races was observed by the comparison of proton spectra. Analyzed spectra showed similar metabolite composition in sugar region from (3 to 5.5 ppm), although variability in sucrose concentration in all studied species was observed. Moreover, the highest variation of citric acid and malic acid was detected in all examined species. The binning integrals of the metabolites were used to build PCA and OPLS-DA models. Grouping trends were visualized by means of PCA, while the separation of samples and evaluation of the possible correlation among metabolites were achieved by OPLS-DA. By means of these methods, ten races were grouped into two main clusters: the first one included four hybrid genotypes, and the second consisted of a free pollination cultivar and five experimental races produced by the National Institute of Forestry, Agriculture and Livestock Research (INIFAP). It was found that the race Colosous belonging to the first cluster was characterized by the presence of ascorbic acid that was not found in any other races. Moreover, the first group displayed a higher concentration of citric acid than the second group. The higher concentrations of metabolites such as asparagine, fumaric acid, GABA, glucose, malic acid, pyruvic, quinic acid, sucrose, and tryptophan were detected in the second group [68].

NMR-based metabolomics was also applied for authentication and quality control of *Curcuma longa* L., commonly known as turmeric. Both root and rhizome are mainly used as a spice and food supplement. The main active component of *Curcuma longa* L. is curcumin, which is responsible for many pharmacological activities such as antioxidant, anti-inflammatory, and antibacterial activity [69]. Unfortunately, the lower price, strong yellow color and a wide availability of other *Curcuma* species lead to adulteration of *Curcuma longa* L. Thus, it is important to check the authenticity of *Curcuma longa* L. in order to ensure the good quality and safety of the products. Windarsih et al. employed ^1^H NMR metabolite fingerprinting to differentiate pure and adulterated powder of *Curcuma longa* L. with *Curcuma heyneana.* For this purpose, rhizomes of both species were collected, cleaned, and ground into powder. Adulterated samples were prepared by mixing *Curcuma longa* L. with *Curcuma heyneana* in various concentrations of adulterant. Prior to NMR analysis sample extraction (25 mg of sample in the mixture of CD_3_OD and D_2_O phosphate buffer) was conducted. All NMR experiments were recorded on a Bruker 500 MHz spectrometer. The chemical composition of extracted samples consisted of amino acids, organic acids, sugars, and aromatic compounds. The metabolic profile of *Curcuma longa* L. showed higher intensities from sugars and aromatic compounds compared to *Curcuma heyneana*. Differentiation between both species was achieved by means of PCA, while OPLS-DA analysis allowed for classification between pure and adulterated samples of *C. longa*. The clear separation was obtained on the basis of sugars and curcuminoids, which proved to be indicators of the authenticity of *Curcuma longa* [70].

### 3.5. Diffusion Ordered Spectroscopy (DOSY)

As mentioned in the previous section, the major challenge for NMR spectroscopy is the analysis of complex mixtures, especially with many overlapped signals. Depending on the degree of spectral overlap, interpretation and quantification can be difficult or even impossible. One of the solutions to this problem is a diffusion NMR experiment, which not only provides comprehensive information on the sample but also gives the possibility of differentiation of compounds having the same chemical moieties and different molecular weights. Diffusion-ordered NMR spectroscopy allows for the separation of components based on differences in their translational diffusion coefficients and in a visualization of data, a pseudo-2 dimensional spectra are obtained where one dimension accounts for chemical shift and the other for diffusion coefficients. To measure self-diffusion coefficients of molecules the pulsed gradient stimulated echo (PGSE) experiment can be performed. A series of spin echo spectra is acquired with different field gradient pulse strength, and the exponential signal decays are analyzed in order to estimate the diffusion coefficient which is directly related to the decay rate. The diffusion coefficient depends on many factors such as molecular weight, size, shape, charge, temperature, and aggregation state. The value of the diffusion coefficient decreases with increasing molecular weight, thus signals from small molecules decay more rapidly than those from large molecules. The results obtained with PGSE are similar to the information from HPLC NMR, in which spectra of chromatography fractions are recorded separately, however PGSE is much simpler because it does not require a physical separation of the mixture. Although DOSY is a promising analytical tool, it has drawbacks for example, in the case of molecules which diffuse at similar rates, the separation in the diffusion dimension can be poor. Generally, in DOSY experiment we can differentiate two areas: low resolution DOSY and high resolution DOSY. The first area refers to simple mixtures with widely differentiating sizes and poorly resolved NMR spectra, and the second concerns on complex mixtures with very similar sizes of molecules but well-resolved spectra. In order to improve resolution in the diffusion dimension the matrix-assisted DOSY methods was proposed. By means of this method relatively small differences of the order of 1% in diffusion coefficient can be resolved. Matrix-assisted DOSY improved diffusion resolution by the addition of a co-solute (e.g., polymer micelles, surfactants), which modulates the diffusion properties of components of interest [52,71]. The selection of the appropriate matrix plays a key role in this technique, due to the fact that the modulation of the observed diffusion coefficient depends on differential interaction between the matrix and analytes. The exchange between free and interacting state should be rapid, because slow exchange leads to an increase in spectral complexity. In addition, improperly selected matrix causes line broadening or leads to additional signals in the spectrum. By means of matrix-assisted DOSY signals from isomers, which have nearly identical diffusion coefficients can be differentiated, while in standard DOSY experiment isomers are indistinguishable [72]. The resolution can be also improved by incorporating an additional dimension such as combination of DOSY-TOCSY, DOSY-NOESY or DOSY-COSY [41].

#### Applications

Although DOSY offers the possibility to distinguish compounds having the same chemical moieties and different molecular weights, its application in spices analysis is not as common as other NMR methods. So far the DOSY technique was successfully employed for Port wine samples of different ages [73], tomato juice [74], mango juice [75], extra virgin olive [76], honey [77], milk composition [78], and cheese [79]. DOSY approach was used to characterize saffron samples of different geographical origin (Greece, Spain, Hungary, Turkey and Italy). Saffron is produced from the dried red stigmas of *Crocus sativus* L. and it is the most expensive spice in the world owing to its harvesting requires work and time. It is impossible to harvest the flowers by machinery because they are too delicate, thus they must be harvested by hand. Moreover, to obtain 1 kg spice, 150,000 flowers must be picked up, due to one stigma of saffron weights about 2 mg. The major producers of saffron are Iran, Morocco, India, Spain, Azerbaijan, Italy, and Greece. Saffron is mainly used as a spice and food colorant. However, due to its anti-inflammatory, sedative, analgesic, and antioxidant properties, it can be used for the treatment of different disorders and as a functional food. The main components of saffron which are responsible for its bioactive properties are crocetin, picrocrocin and safranal. The quantity of these three compounds are used to determine the quality of saffron in accordance to the ISO 3632 standard specifications. Therefore, it is important to define the composition of saffron from different geographical origin to properly determine its quality. For this purpose, Sobolev et al. proposed NMR-based approach, in which the metabolic profile of geographically different saffron extracts was characterized, and a comparison between them was made. Before NMR analysis a microwave-assisted extraction procedure was conducted, which not only reduced the extraction time to 30 min but also lowered the saffron quantity to 10 mg. To observe polar and non-polar metabolites of saffron, methanol was used for extraction. All NMR spectra including ^1^H, ^1^H-^1^H TOCSY, ^1^H-^13^C HSQC, ^1^H-^13^C HMBC, and DOSY were recorded on Bruker Avance 600 MHz spectrometer. The chemical composition of saffron including crocin, picrocrocin, linolenic and linoleic fatty acids, acetic acids, phosphatidylcholine, α- and β-glucose content was determined. Based on the DOSY experiment the presence of all–*trans*-crocin for the aglycone moiety, and glucose as well as gentiobiose were confirmed. The highest value of self-diffusion coefficient of free glucose allowed to confirm its presence. Significant differences in metabolic profile of saffron extracts that related to different origins were found on the basis of PCA. The analysis performed for the intensities of 12 selected ^1^H peaks allow to discriminate the samples according to their geographical origin. Saffron samples derived from Turkey and Hungary were characterized by lack of crocins and picrocrocin, and thus were well separated from other samples. The obtained results indicated that these two samples were not saffron. The highest content of a crocins and picrocrocin was observed for two PDO samples from Arbuzzi and Sardinia, and commercial sample from Latium. The other six samples were placed between these two groups. Due to the fact that the content of crocin and picrocrocin may be directly measured by NMR, this method can be used for assessment of saffron quality [25,80]. More papers about application of NMR approaches to saffron studies were described in review by Consonni et al. [9].

### 3.6. SNIF-NMR Site-Specific Natural Isotope Fractionation

NMR-based isotopic analysis is one of the most widely used methods to track the origin of molecules. Stable isotope analysis at natural abundance was applied in food science, pharmaceutical studies for identification of counterfeit drugs, in metabolic studies, and environmental or forensic studies [81]. Due to the fact that molecules exist in different isotopic compositions, they react differently during physical, chemical or biological processes occurring in living systems. As a consequence, these processes lead to the isotopic fractionation and their products are enriched with the lighter isotope which reacts faster than the heavier isotope. The small, but significant and detectable by NMR variations in the isotope composition of molecules during synthetic or metabolic transformations allows to form a unique isotope profile, which shows the history of the molecule [82].

The most commonly used technique for determining isotope ratio is mass spectrometry (irm-MS, isotope ratio measurement by mass spectrometry). This method requires a small amount of sample, but its main disadvantage is the fact that it destroys the compound. Moreover, by means of irm-MS only the global isotope composition can be determined. An alternative method, which provide isotopic information on each molecular position and is non-destructive, is site-specific natural isotope fractionation (SNIF NMR or irm-NMR, isotope ratio measured by NMR). By means of SNIF-NMR the isotopic ratio for ^2^H, ^13^C and ^15^N can be determined. For the first time ^2^H NMR isotopic experiment was applied by Martin et al. to determine the chemical and geographical origin of molecules and to study the biochemical mechanisms [83]. The authors studied different compounds bearing an ethyl group in their structures. It was noticed that this method had great potential in determining the geographical origin because the deuterium contents of the two ethyl acetate samples, purchased in Europe and America, were different. The researchers were confident that the main factor which determined the changes in deuterium ratio was the chemical history of the compound. Seven years later ^2^H SNIF-NMR became the official method for the detection of sugar addition in wine and for the determination of its origin. Nowadays, this technique is commonly used in food chemistry for authentication of the wine origin, fruit juice and expensive natural products, such as vanillin. Moreover, ^2^H SNIF-NMR was applied in detection of counterfeited drugs and forensic investigation [81]. The major disadvantages of ^2^H SNIF-NMR are low natural abundance (0.015%), small magnetogyric ratio (4.107 × 10^7^ rad/s^−1^·T^−1^), overlapping signals due to small frequency range, the risk of hydrogen exchange and low molecular dynamic ranges due to quadrupolar relaxation. The low sensitivity of ^2^H results in long measurement times and requires high concentration of sample for analysis, which limits this technique to small molecules. However, deuterium is a quadrupolar nucleus and, as a consequence, the nuclear Overhauser effect affecting the signal intensity might be neglected. Determination of the isotopic composition at different molecular sites in ^2^H SNIF-NMR was performed through comparison of the integrals of appropriate isotopomers with the integral of signal from an internal reference with known isotopic composition (e.g., tetramethylurea, TMU).

Apart from irm-^2^H NMR, the isotope ratio ^13^C/^12^C was also applied in authentication studies. The major limitations of ^13^C include low sensitivity due to the low natural abundance (1.1%), low magnetogyric ratio (6.726 × 10^7^ rad/s^−1^·T^−1^), and long relaxation time resulting in long measurement times in order to obtain quantitative results and presence of nuclear Overhauser effect, which impacts on the signal intensities. However, ^13^C SNIF-NMR approach is characterized by higher precision and accuracy (better than 0.1%), and lower amount of material needed for analysis in comparison to ^2^H SNIF-NMR. The duration of the experiment can be reduced by addition of a paramagnetic reagent, for example chromium (III) acetylacetonate, which decreases the T_1_ relaxation time. Data obtained from irm-^13^C NMR measurements serve as a fingerprint because it indicates the isotopic composition of the raw material, types of chemical reaction, the yield of each reaction, and geographical origin. Therefore, this approach is an ideal method for the detection of drug counterfeiting and patent infringement because each process connected with the final product influences the isotopic fingerprint. More details about NMR principles and conditions for isotopic analysis are presented in reviews by Jézéquel et al. [81] and Akoka et al. [82].

#### Applications

The combination of ^2^H and ^13^C SNIF-NMR was applied to study the authenticity of vanillin. Natural vanillin is obtained from the pods of *Vanilla planifolia* (Andrews) orchids produced in smallholder farms in Madagascar and its price is very high in comparison to its synthetic alternative. Moreover, *Vanilla planifolia* is grown in Indonesia, Mauritius, and Comoros, but only the species from Madagascar are protected by the Bourbon label since 1964. It is estimated that vanilla is the second most expensive spice in the world right after saffron. To obtain 1 kg of pure vanillin, 50 kg of pods is required because a pod contains only about 2% of vanillin. Vanillin can be also obtained via biosynthesis by using natural precursors such as ferulic acid extracted from rice and maize, eugenol extracted from clove oil, and curcumin extracted from curcuma. Natural vanillin is adulterated by synthetic vanillin manufactured from guaiacol derived from petrol derivatives or by semi-synthetic vanillin produced from lignin, coming from paper residues. About 85% of the annual production of vanillin is derived from guaiacol, less than 1% from natural sources and most of the rest from lignin [84]. In the case of vanillin two different types of fraud are distinguishable: (1) substitution of natural vanillin by synthetic and (2) mislabeling, where synthetic vanillin is assigned as natural. Natural vanillin was characterized by crassulacean acid metabolism photosynthesis (CAM), which is typical for plants adapted to very dry conditions, thus its isotopic composition may be used for determination of geographical origin. The official method for authentication of vanillin—irm-^13^C MS indicated that natural vanillin is characterized by δ^13^C equal −20‰, while for vanillin of unnatural origin δ^13^C is in the range from −29‰ to −31‰. However, the addition of commercially available ^13^C-enriched vanillin leads to compensation of this difference and irm-^13^C MS became much less effective. Thus, the detection of fraud should be performed on the basis of specific enrichment on one or several sites of the vanillin molecule, which is possible by means of ^2^H and ^13^C SNIF-NMR. As mentioned above, the main disadvantage of ^2^H SNIF-NMR is low natural abundance of deuterium, which results in the requirement of long duration of measurement—in the case of vanillin around 15 h to obtain three spectra and high amount of compound for analysis (1 g of pure vanillin). Contrary to ^2^H, the amount of 250 mg of pure vanillin is necessary for the ^13^C SNIF-NMR analysis. Furthermore, the analysis time is reduced to less than 8 h for five spectra. Remaud et al. conducted a study in which the data obtained from both methods were compared and their utility for determination of the origin of vanillin was checked. The analysis was conducted for 65 authentic samples of vanillin: 8 from *V. planifolia* beans from Bourbon origin, and 3 from Indonesia. Forty samples derived from biosynthesis: 31 vanillin samples extracted from ferulic acid from rice, 1 from ex-ferulic acid from maize, 3 from ex-curcumin, 4 from ex-eugenol, 1 from ex-isoeugenol and 14 synthetic samples (12 from ex-guaiacol and 2 from ex-lignin). Due to too little amount of product, 17 samples were not analyzed by ^2^H and 7 samples were not analyzed by means of ^13^C SNIF-NMR. For ^2^H, SNIF NMR samples were prepared by weighing 1.05 g of vanillin, 0.22 mL of TMU, 1.55 mL of acetonitrile, 0.10 mL of C_6_F_6_, 0.05 mL of trifluoroacetic acid. Then, the mixture was heated to dissolve vanillin and transferred through a 0.45 μm filter to a 10 mm NMR tube. In the case of ^13^C SNIF-NMR 250 mg of vanillin with 400 μL of acetone-d5 and 100 μL of relaxation agent Cr(acac)_3_ solution (0.1M, in order to perform spectra in a reasonable time-frame) were mixed and then filtered through a 0.45 μm filter to 5 mm NMR tube. All ^2^H measurements were conducted on Bruker Avance III 400 MHz NMR spectrometer, equipped with a 10 mm SEX ^2^H/^1^H probe with a ^19^F locking device, while ^13^C were performed on an Avance I 400 MHz equipped with 5 mm ^1^H/^13^C dual+ probe (Bruker, Biospin, Wissembourg, France). By means of ^2^H SNIF-NMR the isotopic composition for the four resolved ^2^H isotopomers were determined and their discrimination power were checked by means of PCA analysis. Due to the exchangeable character of the hydroxyl group this position was excluded from analysis. PCA analysis conducted for deuterium isotopic composition at four sites allow for good separation of vanillin derived from three origins: ex-guaiacol, ex-ferulic acid from rice, and ex-beans samples. Deuterium analysis were not able to distinguish vanillin ex-beans samples from Indonesia from vanillin samples with a Bourbon label. Moreover, two distinct groups for vanillin derived from ex-guaiacol was observed. This differentiation was explained by a different type of the glyoxylic acid used for synthesis of vanillin (one glyoxylic acid derived from acetaldehyde and the other from maleic anhydride). The difference obtained for ^2^H content of site 1 of vanillin was more than 200 ppm, when two distinct sources of glyoxylic acid were used in the same process. In the case of ^13^C SNIF-NMR for PCA analysis, eight well-separated isotopomers were used. In comparison to results obtained for ^2^H isotopic composition on the basis of ^13^C isotopic profiles all origins of vanillin were distinguished by precursor, including the geographical origin of vanilla beans. Because each of the isotopic profiles provides information, which explain the variability between the samples, to improve the distinction of samples data fusion approach was applied. Two PCA matrices obtained for isotopic compositions of ^2^H and ^13^C were applied to build the classification model, however obtained results did not improve differentiation of samples. The authors found that ^13^C SNIF-NMR was the most precise tool and had better potential of origin detection. In the case of ^2^H isotopic composition there is a 20% risk to inappropriate classification of ex-guaiacol vanillin as a natural (96% accuracy, 80% specificity). The verification of naturalness of vanillin by means of ^13^C isotopic composition were characterized by 100% accuracy, precision, sensibility, and specificity for each group. Moreover, the obtained for ^13^C SNIF-NMR model was verified by experiment, in which three mixtures with different content of vanillin precursors were prepared. The first mixture consisted of 75% of ferulic acid from rice and 25% of guaiacol, the second 50% of ferulic acid from rice and 50% of guaiacol, and the last consisted of 50% of ferulic acid from rice and 50% of vanilla pods. All mixtures were correctly discriminated. In addition, the amount of sample for measurements was significantly reduced (30 mg) in comparison to deuterium measurements, when INEPT (insensitive nuclei enhanced by polarization transfer) pulse sequence was used. Due to magnetization transfer from the sensitive proton to the coupled carbon an improvement in the sensitivity was achieved. Furthermore, by means of INEPT the precision of the order of 1‰ was able to be reached [82,85].

NMR approaches used in the adulteration of spices were summarized in Table 6.

## 4. Conclusions

This review demonstrates that NMR spectroscopy combined with a chemometric approach constitutes an efficient tool for the quality assessment and authentication of spices. The progress made in the field of NMR spectroscopy towards improving the sensitivity and resolution enables rapid identification of compounds in complex mixtures without the need for prior separation of components. The different NMR approaches offer an excellent comprehensive method for the characterization, quantification and discrimination between samples, which guarantee their safety and quality as well as sustainability. Moreover, by applying the HR–MAS–NMR technique, analysis may be conducted without any chemical treatment, which ensures its biological and chemical integrity. Furthermore, the development of hybrid techniques such as LC–SPE–NMR and sensing methods for NMR-based detection has greatly increased the analytical capabilities in the comprehensive analysis of small molecules from complex matrices [86,87,88,89]. According to our knowledge, the molecular sensors have not yet been employed in the analysis of spices, although in our opinion their application is only a matter of time. In the future, the major challenge will be the development of portable devices operating at frequencies higher than 100 MHz, which may not only serve as an important tool for fraud detection but also may be helpful in food processing at the industrial level. Moreover, the continuous development of chemometrics as data fusion and big data approaches to observe the maximum information contained in the spectral data is required.

## Figures and Tables

**Table 1:** Number of papers about using spectroscopic methods in spices adulteration and quality control.

Spectroscopic Method	Number of Records *	Number of Records **	Number of Records ***
**NMR**	29	5	250
**Raman**	3	8	83
**NIR**	21	5	54
**FT-IR**	18	4	127

*—query: spices control (spectroscopic method). **—query: spices authentication (spectroscopic method). ***—query: spices (spectroscopic method).

**Table 2:** Differences in the metabolites contents with respect to geographical origin of red pepper powders.

Metabolite	Red Pepper Powders		
	Vietnam	China	Korea
Tyrosine	higher content (10×)		
Alanine	higher content		
α- and β-glucose			higher content
Tryptophan			higher content
Adenosine			higher content
Kaempferol		lower content	

**Table 3:** Comparison of typical parameters for standard and quantitative measurement conditions.

Parameters	Standard NMR	qNMR
Repetition Time	~5 s	>T_1_ × 7
Acquisition Time	2 s	2–6 s
Pulse Flip Angle	30–50°	90°
Number of Scans	8	S/N > 150
Digital Resolution	0.5 Hz	<0.2 Hz
^13^C Decoupling	Off	On
Sample Spinning	On	Off

**Table 4:** Differences in the metabolites contents with respect to the three Italian *A. cepa* L. cultivars.

**Metabolites**	**Fresh Onion Bulbs**		
	**White Onion, WP**	**Red Onion, RT**	**Yellow Onion, CM**
Citrate		higher content	
Glucose	higher content	higher content	
Sucrose	higher content		
FOS	higher content		
Methiin			higher content
Free isoalliin			higher content
γ-glutamyl-isoalliin			higher content
Glutamine			higher content
Malate			higher content
Sterols	higher content		
Choline			higher content
**Metabolites**	**Stored Onion Bulbs**		
	**White Onion, WPS**	**Red Onion, RTS**	**Yellow Onion, CMS**
2-carboxilpropyl cysteine	higher content		
Glucose	higher content		
Sucrose	higher content		
FOS	higher content		
Valine	higher content		
Leucine	higher content		
Isoleucine	higher content		
GABA			higher content
Asparagine			higher content
Methiin			higher content
Free isoalliin			higher content
Glutamate			higher content
Glutamine			higher content
Tyrosine			higher content
Sterols	higher content		
Cycloartenol		traces	
Cycloalliin		higher content	

**Table 5:** Differences in the metabolites contents with respect to geographical origin of Serrano peppers.

Metabolites	Serrano Peppers	
	Veracruz	Oaxaca
Aspartate	higher content	
Citrate	higher content	
Lactate	higher content	
Leucine	higher content	
Sucrose	higher content	
Acetate		higher content
Formate		higher content
Fumarate		higher content
Malonate		higher content
Phosphocholine		higher content
Pyruvate		higher content
Succinate		higher content

**Table 6:** A summary overview of NMR approaches used in the adulteration of spices.

Spices	NMR Approach	Chemometric Approach	References
Black Pepper	^1^H, ^13^C NMR	-	[47]
Chili Peppers	^1^H NMR-based metabolomics	PCA, LDA, HCA	[45,67]
Cinnamon	qNMR	PCA, OPLS-DA	[54]
Coriander	^1^H NMR F_1_-PSYCHE-TOCSY	-	[48]
Garlic	^1^H NMR, qNMR ^1^H HRMAS NMR	PCA, PLS-DA	[57,58]
Guinea Pepper	^1^H NMR, 2D NMR,	-	[49]
Japaleño Peppers	^1^H NMR-based metabolomics	PCA, OPLS-DA, ANOVA	[68]
Onion	^1^H NMR, qNMR, ^1^H HRMAS NMR, ^1^H NMR-based metabolomics	PCA, PLS-DA, OPLS-DA	[58,65]
Paprika Powder	^1^H NMR, qNMR ^1^H HRMAS NMR	-	[56]
Red Pepper Powder	^1^H NMR, ^1^H HRMAS NMR	ANOVA, canonical discriminant analysis, PLS-DA	[43,44,62]
Saffron	^1^H NMR, 2D NMR, DOSY	PCA	[80]
Turmeric	^1^H NMR-based metabolomics	PCA, OPLS-DA	[70]
Vanillin	^2^H SNIF-NMR ^13^C SNIF-NMR	PCA	[85]

## References

[1] Spices & Culinary Herbs. https://www.statista.com/outlook/40070300/100/spices-culinary-herbs/worldwide.

[2] Global Seasonings & Spices Market Size Report, 2020–2027. https://www.grandviewresearch.com/industry-analysis/seasonings-spices-market.

[3] Muggeridge M., Clay M., Peter K.V. (2001). Quality specifications for herbs and spices. Handbook of Herbs and Spices.

[4] Europol Over €100 Million Worth of Fake Food and Drinks Seized in Latest Europol-Interpol Operation. https://www.europol.europa.eu/newsroom/news/over-€100-million-worth-of-fake-food-and-drinks-seized-in-latest-europol-interpol-operation.

[5] Kucharska-Ambrożej K., Karpińska J. (2020). The application of spectroscopic techniques in combination with chemometrics for detection adulteration of some herbs and spices. Microchem. J..

[6] Spink J., Moyer D.C. (2011). Defining the Public Health Threat of Food Fraud. J. Food Sci..

[7] Moore J.C., Spink J., Lipp M. (2012). Development and Application of a Database of Food Ingredient Fraud and Economically Motivated Adulteration from 1980 to 2010. J. Food Sci..

[8] USP Food Fraud Database, Glossary of Terms. http://www.foodfraud.org/glossary-terms.

[9] Consonni R., Cagliani L.R. (2019). The potentiality of NMR-based metabolomics in food science and food authentication assessment. Magn. Reson. Chem..

[10] Oliviera M.M., Cruz-Tirado J.P., Barbin D.F. (2019). Nontargeted analytical methods as a powerful tool for the authentication of spices and herbs: A review. Compr. Rev. Food Sci. Food Saf..

[11] Everstine K., Spink J., Kennedy S. (2013). Economically Motivated Adulteration (EMA) of Food: Common Characteristics of EMA Incidents. J. Food Prot..

[12] Lakshmi V., Pradesh A. (2012). Food adulteration. Int. J. Sci. Invent. Today.

[13] Everstine K. (2013). Economically Motivated Adulteration: Implications for Food Protection and Alternate Approaches to Detection. Ph.D. Thesis.

[14] Ellis D.I., Brewster V.L., Dunn W.B., Allwood J.W., Golovanov A.P., Goodacre R. (2012). Fingerprinting food: Current technologies for the detection of food adulteration and contamination. Chem. Soc. Rev..

[15] Lohumi S., Lee S., Lee H., Cho B.-K. (2015). A review of vibrational spectroscopic techniques for the detection of food authenticity and adulteration. Trends Food Sci. Technol..

[16] Nisa A., Zahra N., Butt Y.N. (2016). Sudan dyes and their potential health effects. Pak. J. Biochem. Mol. Biol..

[17] Contreras-Castillo C.J., Cruz-Tirado J.P., Shirahigue L.D., Rodriguez J.M.L., Ruiz D.F. (2016). The science of grape seeds applied to health food development. Grape Seeds: Nutrient Content, Antioxidant Properties and Health Benefits.

[18] Blythman J. (2005). Hidden secrets of the colourful spice market. The Evening Standard.

[19] Cheng Y.-Y., Tsai T.-H. (2016). A Validated LC–MS/MS Determination Method for the Illegal Food Additive Rhodamine B: Applications of a Pharmacokinetic Study in Rats. J. Pharm. Biomed. Anal..

[20] Mohiuddin A.K. (2020). Health hazards with adulterated spices: Save the “Onion Tears”. Innov. J. Med. Sci..

[21] Nallappan K., Dash J., Ray S., Pesala B. Identification of adulterants in turmeric powder using terahertz spectroscopy. Proceedings of the 38th International Conference on Infrared, Millimeter, and Terahertz Waves (IRMMW-THz).

[22] Mohiuddin A.K. (2019). Chemical Contaminants and Pollutants in the Measurable Life of Dhaka City. Glob. J. Pharm. Sci..

[23] Petrakis E.A., Polissiou M.G. (2017). Assessing saffron (*Crocus sativus* L.) adulteration with plant-derived adulterants by diffuse reflectance infrared Fourier transform spectroscopy coupled with chemometrics. Talanta.

[24] European Commission RASFF Annual Report 2019. https://op.europa.eu/en/publication-detail/-/publication/2c5c7729-0c31-11eb-bc07-01aa75ed71a1/language-en/format-PDF/source-174742448.

[25] Nazari S.H., Kefi N. (2007). Saffron and various fraud manners in its production and trades. Acta Hortic..

[26] Monakhova Y.B., Tsikin A.M., Kuballa T., Lachenmeier D.W., Mushtakova S.P. (2014). Independent component analysis (ICA) algorithms for improved spectral deconvolution of overlapped signals in ^1^H NMR analysis: Application to foods and related products. Magn. Reson. Chem..

[27] Yoshitomi T., Wakana D., Uchiyama N., Tsujimoto T., Kawano N., Yokokura T., Yamamoto Y., Fuchino H., Hakamatsuka T., Komatsu K. (2020). ^1^H NMR-based metabolomic analysis coupled with reversed-phase solid-phase extraction for sample preparation of Saposhnikovia roots and related crude drugs. J. Nat. Med..

[28] Cook D.W., Rutan S.C. (2014). Chemometrics for the analysis of chromatographic date in metabolomics investigations. J. Chemometr..

[29] Defernez M., Colquhoun I.J. (2003). Factors affecting the robustness of metabolite fingerprinting using ^1^H NMR spectra. Phytochemistry.

[30] Hatzakiz E. (2019). Nuclear Magnetic Resonance (NMR) Spectroscopy in Food Science: A Comprehensive Review. Compr. Rev. Food Sci. Food Saf..

[31] Craig A., Cloarec O., Holmes E., Nicholson J.K., Lindon J.C. (2006). Scaling and normalization effects in NMR spectroscopic metabonomic data sets. Anal. Chem..

[32] Krakowska B., Custers D., Deconinck E., Daszykowski M. (2016). Chemometrics and the identification of counterfeit medicines—A review. J. Pharm. Biomed. Anal..

[33] Domingue J., Lasierra N., Fensel A., van Kasteren T., Strohbach M., Thalhammer A., Cavanillas J., Curry E., Wahlster W. (2016). Big Data Analysis. New Horizons for a Data-Driven Economy.

[34] Van der Kooy F., Maltese F., Choi Y.H., Kim H.K., Verpoorte R. (2009). Quality control of herbal material and phytopharmaceuticals with MS and NMR based metabolic fingerprinting. Planta Med..

[35] Le Gall G., Colquhoun I.J., Lees M. (2003). NMR spectroscopy in food authentication. Food Authenticity and Traceability.

[36] Parlak Y., Güzeler N. (2016). Nuclear Magnetic Resonance Spectroscopy Applications in Foods. Curr. Res. Nutr. Food Sci..

[37] Corsaro C., Cicero N., Mallamace D., Vasi S., Naccari C., Salvo A., Giofrè S.V., Dugo G. (2016). HR-MAS and NMR towards Foodomics. Food Res. Int..

[38] Zangger K. (2015). Pure shift NMR. Prog. Nucl. Magn. Reson. Spectrosc..

[39] Foroozandeh M., Morris G.A., Nilsson M. (2018). PSYCHE Pure Shift NMR Spectroscopy. Chem. Eur. J..

[40] Nolis P., Motiram-Corral K., Pérez-Trujillo M., Parella T. (2019). Interleaved Dual NMR Acquisition of Equivalent Transfer Pathways in TOCSY and HSQC Experiments. Chem. Phys. Chem..

[41] Barjat H., Morris G.A., Swanson A.G. (1998). A three-dimensional DOSY-HMQC experiment for the high-resolution analysis of complex mixtures. J. Magn. Reson..

[42] Gallo V., Ragone R., Musio B., Todisco S., Rizzuti A., Mastrorilli P., Pontrelli S., Intini N., Scapicchio P., Triggiani M. (2020). A contribution to the harmonization of non-targeted NMR methods for data-driven food authenticity assessment. Food Anal. Methods.

[43] Lee D., Kim M., Kim B.H., Ahn S. (2020). Identification of the Geographical Origin of Asian Red Pepper (*Capsicum annuum* L.) Powders Using ^1^H NMR spectroscopy. Bull. Korean Chem. Soc..

[44] Sobolev A.P., Mannina L., Capitani D., Sanzò G., Ingallina C., Botta B., Fornarini S., Crestoni M.E., Chiavarino B., Carradori S. (2018). A multi-methodological approach in the study of Italian PDO “Cornetto di Pontecorvo” red sweet pepper. Food Chem..

[45] Al-Samydai A., Aburjai T., Alshaer W., Azzam H., Al-Mamoori F. (2019). Qualitative and Quantitative Analysis of Capsaicin from Capsicum annum Grown in Jordan. Int. J. Res. Pharm. Sci..

[46] Islam M.S., Noor M.A., Hossain M.S., Saha K.C., Seal H. (2015). Chemical investigation of bioactive compounds of black pepper. Int. J. Pharm. Sci. Res..

[47] Ahmad R., Ahmad N., Amir M., Aljishi F., Alamer M.H., Al-Shaban H.R., Alsadah Z.A., Alsultan B.M., Aldawood N.A., Chathoth S. (2020). Quality variation and standardization of black pepper (*Piper nigrum*): A comparative geographical evaluation based on instrumental and metabolomics analysis. Biomed. Chromatogr..

[48] Verma A., Parihar R., Baishya B. (2019). Identification of metabolites in coriander seeds (*Coriandrum Sativum* L.) aided by ultrahigh resolution total correlation NMR spectroscopy. Magn. Reson. Chem..

[49] Zhang Z., Zulfiqar F., Zulfiqar A., Kahn I.A. (2020). Two undescribed paradol-related specialized metabolites from *Aframomum melegueta*. Nat. Prod. Res..

[50] Karakaya S., Simsek D., Ozbek H., Guvenlap Z., Altanlar N., Kazaz C., Kilic C.S. (2019). Antimicrobial activities of extracts and isolated coumarins from the roots of four *Ferulago* species growing in Turkey. Iran. J. Pharm. Res..

[51] Bharti S.K., Roy R. (2012). Quantitative ^1^H NMR spectroscopy. Trends Analyt. Chem..

[52] Diehl B., Holzgrabe U., Monakhova Y., Schönberger T. (2020). Quo Vadis qNMR?. J. Pharm. Biomed. Anal..

[53] Pauli G.F., Jaki B.U., Lankin D.C. (2005). Quantitative ^1^H NMR: Development and Potential of a Method for Natural Products Analysis. J. Nat. Prod..

[54] Farag M.A., Labib R.M., Noleto C., Porzel A., Wessjohann L.A. (2018). NMR approach for the authentication of 10 cinnamon spice accessions analyzed via chemometric tools. LWT.

[55] Villa-Ruano N., Ramírez-Meraz M., Méndez-Aguilar R., Zepeda G., Alvarez bravo A., Pérez-Hernández N., Becerra-Martinez E. (2018). ^1^H NMR-based metabolomics profiling of ten new races from *Capsicum annuum* cv. serrano produced in Mexico. Food Res. Int..

[56] Hu Y., Wang S., Wang S., Lu X. (2017). Application of nuclear magnetic resonance spectroscopy in food adulteration determination: The example of Sudan dye 1 in paprika powder. Sci. Rep..

[57] Ritota M., Casciani L., Han B.-Z., Cozzolino S., Leita L., Sequi P., Valentini M. (2012). Traceability of Italian garlic (*Allium sativum* L.) by means of HRMAS-NMR spectroscopy and multivariate data analysis. Food Chem..

[58] Jo S., Song Y., Jeong J.-H., Hwang J., Kim Y. (2020). Geographical discrimination of *Allium* species (garlic and onion) using ^1^H NMR spectroscopy with multivariate analysis. Int. J. Food Prop..

[59] Andrew E.R., Newing R.A. (1958). The Narrowing of Nuclear Magnetic Resonance Spectra by Molecular Rotation in Solids. Proc. Phys. Soc..

[60] Mazzei P., Piccolo A. (2017). HRMAS NMR spectroscopy applications in agriculture. Chem. Biol. Technol. Agric..

[61] Corsaro C., Mallamace D., Vasi S., Ferrantelli V., Dugo G., Cicero N. (2015). ^1^H HR-MAS NMR Spectroscopy and the Metabolite Determination of Typical Foods in Mediterranean Diet. J. Anal. Methods Chem..

[62] Ritota M., Marini F., Sequi P., Valentini M. (2010). Metabolomic Characterization of Italian Sweet Pepper (*Capsicum annum* L.) by Means of HRMAS-NMR Spectroscopy and Multivariate Analysis. J. Agric. Food Chem..

[63] Sattar S., Das P.C., Hossain M.S., Sarower K., Uddin M.B. (2019). Study on Consumer Perception towards Quality of Spices Powder Available in Bangladesh. Open J. Saf. Sci. Technol..

[64] Markley J.L., Brüschweiler R., Edison A.S., Eghbalnia H.R., Powers R., Raftery D., Wishart D.S. (2017). The future of NMR-based metabolomics. Curr. Opin. Biotechnol..

[65] Saviano G., Paris D., Melck D., Fantasma F., Motta A., Iorizzi M. (2019). Metabolite variation in three edible Italian *Allium cepa* L. by NMR-based metabolomics: A comparative study in fresh and stored bulbs. Metabolomics.

[66] Becerra-Martinez E., Florentino-Ramos E., Pérez-Hernández N., Zepeda-Vallejo L.G., Villa-Ruano N., Velázquez-Ponce M., García-Mendoza F., Bañuelos-Hernández A.E. (2017). ^1^H NMR-based metabolomics fingerprinting to determine metabolite levels in serrano peppers (*Capsicum annum* L.) grown in two different regions. Food Res. Int..

[67] Florentino-Ramos E., Villa-Ruano N., Hidalgo-Martínez D., Ramírez-Meraz M., Méndez-Aguilar R., Velásquez-Valle R., Zepeda-Vallejo L.G., Pérez-Hernández N., Becerra-Martínez E. (2019). ^1^H NMR-based fingerprinting of eleven Mexican *Capsicum annum* cultivars. Food Res. Int..

[68] Ramírez-Meraz M., Méndez-Aguilar R., Hidalgo-Martínez D., Villa-Ruano N., Zepeda-Vallejo L.G., Vallejo-Contreras F., Hernández-Guerrero C.J., Becerra-Martínez E. (2020). Experimental races of *Capsicum annuum* cv. japaleño: Chemical characterization and classification by ^1^H NMR/machine learning. Food Res. Int..

[69] Chatzinasiou L., Booker A., MacLennan E., Mackonochie M., Heinrich M. (2019). Turmeric (*Curcuma longa* L.) products: What quality differences exist?. J. Herb. Med..

[70] Windarsih A., Rohman A., Swasono R.T. (2019). Application of ^1^H-NMR based metabolite fingerprinting and chemometrics for authentication of *Curcuma longa* adulterated with *C. heyeana*. J. Appl. Res. Med. Aroma..

[71] Day I.J. (2019). Matrix-assisted DOSY. Prog. Nucl. Magn. Reson. Spectrosc..

[72] Tormena C.F., Evans E., Haiber S., Nilsson M., Morris G.A. (2012). Matrix-assisted diffusion-ordered spectroscopy: Application of surfactant solutions to the resolution of isomer spectra. Magn. Reson. Chem..

[73] Nilsson M., Duarte I.F., Almeida C., Delgadillo I., Goodfellow B.J., Gil A.M., Morris G.A. (2004). High-Resolution NMR and Diffusion-Ordered Spectroscopy of Port Wine. J. Agric. Food Chem..

[74] Sobolev A.P., Segre A., Lamanna R. (2003). Proton high-field NMR study of tomato juice. Magn. Reson. Chem..

[75] Duarte I.F., Goodfellow B.J., Gil A.M., Delgadillo I. (2005). Characterization of Mango Juice by High-Resolution NMR, Hyphenated NMR, and Diffusion-Ordered Spectroscopy. Spectrosc. Lett..

[76] Demisli S., Theochari I., Christodoulou P., Zervou M., Xenakis A., Papadimitriou V. (2020). Structure, activity and dynamics of extra virgin olive oil-in-water nanoemulsions loaded with vitamin D_3_ and calcium citrate. J. Mol. Liq..

[77] Gresley A.L., Kenny J., Cassar C., Kelly A., Sinclair A., Fielder M.D. (2012). The application of high resolution diffusion NMR to the analysis of Manuka honey. Food Chem..

[78] Tomassini A., Curone G., Solè M., Capuani G., Sciubba F., Conta G., Miccheli A., Vigo D. (2019). NMR-based metabolomics to evaluate the milk composition from Friesian and autochthonous cows of Northern Italy at different lactation times. Nat. Prod. Res..

[79] Bordoni A., Picone G., Babini E., Vignali M., Danesi F., Valli V., Di Nunzio M., Laghi L., Capozzi F. (2011). NMR comparison of *in vitro* digestion of *Parmigiano Reggiano* cheese aged 15 and 30 months. Magn. Reson. Chem..

[80] Sobolev A.P., Carradori S., Capitani D., Vista S., Trella A., Marini F., Manninaet L. (2014). Saffron samples of different origin: An NMR study of microwave-assisted extracts. Foods.

[81] Jézéquel T., Joubert V., Giraudeau P., Remaud G.S., Akoka S. (2017). The new face of isotopic NMR at natural abundance. Magn. Reson. Chem..

[82] Akoka S., Remaud G.S. (2020). NMR-based isotopic and isotopomic analysis. Prog. Nucl. Magn. Reson. Spectrosc..

[83] Martin G.J., Martin M.L. (1981). Deuterium labelling at the natural abundance level as studied by high field quantitative ^2^H NMR. Tetrahedron Lett..

[84] Bomgardner M.M. (2016). The problem with vanilla. Chem. Eng. News.

[85] Guyader S., Thomas F., Jamin E., Grand M., Akoka S., Silvestre V., Remaud G.S. (2019). Combination of ^13^C and ^2^H SNIF-NMR isotopic fingerprints of vanillin to control its precursors. Flavour Fragr. J..

[86] Gathugu R.M., Kautz R., Kristal B.S., Bird S.S., Vouros P. (2018). The integration of LC-MS and NMR for the analysis of low molecular weight trace analytes in complex matrices. Mass Spec. Rev..

[87] Sahu A., Balhara A., Singh D.K., Kataria Y., Singh S. (2018). NMR Spectroscopy, Techniques, LC-NMR and LC-NMR-MS. Encyclopedia of Analytical Science.

[88] Xu Z., Liu C., Zhao S., Chen S., Zhao Y. (2019). Molecular Sensors for NMR-Based Detection. Chem. Rev..

[89] Hermkens N.K.J., Eshuis N., van Weerdenburg B.J.A., Feiters M.C., Rutjes F.P.J.T., Wijmenga S.S., Tessari M. (2016). NMR-based Chemosensing via p-H2 Hyperpolarization: Application to Natural Extracts. Anal. Chem..

